# GnRH-receptor antagonism as a targeted approach to reproductive dysfunction in polycystic ovary syndrome

**DOI:** 10.1016/j.ebiom.2026.106398

**Published:** 2026-07-17

**Authors:** Ludovica Cotellessa, Hélène Maitre, Frank Giton, Silvia Bongiovanni, Pascal Pigny, Geoffroy Robin, Sophie Catteau-Jonard, Paolo Giacobini

**Affiliations:** a Inserm, CHU Lille, Lille Neuroscience & Cognition, UMR-S 1172, Univ. Lille, Lille, France; b AP-HP, Pôle Biologie-Pathologie Henri Mondor, 94010, Créteil, France; c Inserm IMRB U955, 94010, Créteil, France; d CHU Lille, Service de Biochimie et Hormonologie, Centre de Biologie Pathologie, Lille, France; e CHU Lille, Service de Gynécologie Médicale, Hôpital Jeanne de Flandre, Lille, France

**Keywords:** Polycystic ovary syndrome (PCOS), Polyendocrine metabolic ovarian syndrome (PMOS), GnRH, LH, Ganirelix, Fertility, Hyperandrogenism

## Abstract

**Background:**

Polycystic ovary syndrome (PCOS), recently renamed polyendocrine metabolic ovarian syndrome (PMOS), is characterised by neuroendocrine dysfunction with accelerated gonadotrophin-releasing hormone (GnRH)/luteinising hormone (LH) pulsatility driving hyperandrogenism and anovulatory infertility.

**Methods:**

We used the prenatal anti-Müllerian hormone (AMH)-exposed PMOS-like mouse model (PAMH) and a phase I clinical trial in women with PMOS without obesity. PAMH and control mice received acute or intermittent low-dose Ganirelix, and oestrous cyclicity, ovulation, gonadotrophins, and steroids were assessed. In women, two subtherapeutic Ganirelix doses (0.025 mg, *n* = 8; 0.0625 mg, *n* = 10) were administered once in early follicular phase, with 10-min blood sampling over 8 h to quantify LH pulsatility and reproductive hormones.

**Findings:**

In PMOS-like mice, a single Ganirelix injection normalised exaggerated LH pulsatility, and six-week intermittent treatment restored oestrous cyclicity, ovulation, and testosterone levels without affecting controls. In women with PMOS both Ganirelix doses reduced LH pulse frequency, basal, mean and total LH, and decreased the LH/FSH ratio. D4-androstenedione fell by 25–30% at both doses, AMH declined modestly at 0.0625 mg, while oestradiol remained unchanged.

**Interpretation:**

Low-dose GnRH-receptor antagonism with Ganirelix can recalibrate, rather than suppress, GnRH/LH signalling, attenuating hyperandrogenism and, in mice, restoring ovulatory function. These data identify partial GnRHR blockade as a promising neuroendocrine-centred strategy in PMOS and provide a rationale for phase II trials evaluating repeated low-dose regimens, ovulatory restoration, and fertility outcomes.

**Funding:**

This work was supported by the European Research Council (ERC) Horizon-ERC-POC grant (ERC-2022-POC2, n° 101111874) and the French National Research Agency (ANR-24-CHBS-0002, France 2030).

**Research in context**

**Evidence before this study**

PMOS, formerly named PCOS, is marked by increased frequency of signals from the brain that stimulate the release of LH, leading to excess ovarian androgen production and failure to ovulate regularly. While GnRH receptor antagonists are well established in fertility medicine, they are typically administered at doses high enough to completely shut down LH secretion and block ovulation. Because of this, they have not been considered as therapeutic options for PMOS outside assisted reproduction. Previous clinical studies with related antagonists either used doses that were too high to preserve normal reproductive function or did not examine LH pulse patterns. No previous study had systematically evaluated whether low-dose GnRH receptor antagonism could selectively attenuate abnormal LH pulsatility and thereby reduce androgen levels.

**Added value of this study**

This study demonstrates that low-dose Ganirelix can normalise LH pulse dynamics in both a validated PMOS-like mouse model and in women with PMOS. In mice, intermittent low-dose treatment restored hormone balance, oestrous cyclicity, and ovulation. In women, single subtherapeutic doses reduced LH pulse frequency, improved the LH/FSH ratio, and lowered circulating androgens. These results provide evidence that partial GnRH receptor blockade may help correct the core neuroendocrine abnormality underlying PMOS.

**Implications of all the available evidence**

Collectively, these findings provide a foundation for future studies investigating therapeutic strategies aimed at recalibrating, rather than suppressing, GnRH/LH hormone signalling in PMOS. While the current human data establish short-term proof-of-mechanism, confirming that the predicted selective neuroendocrine signature is achievable in women with PMOS, dedicated prospective trials of appropriate duration and design will be required to determine effects on ovulatory restoration, cycle normalisation, and reproductive outcomes.

## Introduction

PCOS, recently renamed polyendocrine metabolic ovarian syndrome (PMOS),^1^ is a prevalent and heterogeneous reproductive and metabolic disorder and the leading cause of anovulatory infertility among women of reproductive age.^2^, ^3^, ^4^ Because the aetiology of PMOS remains largely unknown, no curative treatment is currently available, and patient management remains suboptimal, focussing primarily on symptomatic relief.^2^^,^^3^ Consequently, the syndrome has a substantial impact on the quality of life of affected women.^5^, ^6^, ^7^, ^8^, ^9^ There is therefore an urgent need to develop effective therapeutic strategies aimed at curing and/or reducing the health-related burden associated with PMOS.

The international evidence-based guideline for the assessment and management of PCOS/PMOS endorses the Rotterdam diagnostic criteria.^9^ According to these criteria, diagnosis requires the presence of at least two of the following three features: oligo-anovulation, biochemical or clinical hyperandrogenism, and polycystic ovarian morphology (PCOM) or elevated circulating anti-Müllerian hormone (AMH) levels, after exclusion of other causes of anovulation and hyperandrogenism, including thyroid dysfunction, hyperprolactinemia, congenital adrenal hyperplasia, Cushing syndrome, androgen-secreting tumours, and hypogonadotropic hypogonadism.^4^^,^^9^

A key neuroendocrine abnormality in women with PMOS is an increased LH pulse frequency, observed in approximately 70–75% of cases irrespective of body mass index.^10^, ^11^, ^12^, ^13^, ^14^, ^15^, ^16^ Moreover, up to 90% of women with PMOS exhibit a persistently elevated LH-to-follicle-stimulating hormone (FSH) ratio.^10^, ^11^, ^12^, ^13^, ^14^, ^15^^,^^17^ Together, these observations strongly support the presence of accelerated GnRH pulsatility, as GnRH and LH secretion display a near one-to-one relationship, allowing peripheral LH measurements to serve as a reliable surrogate marker of GnRH neuronal activity.^18^^,^^19^

GnRH neurons are the master regulators of reproductive functions in mammals as they control fertility by driving the secretion of LH and FSH from the pituitary gland. These factors regulate the development and functions of the gonads across vertebrate species.^20^ In women with PMOS, LH hyperpulsatility contributes to increased ovarian thecal androgen production and oligo-anovulation, thereby playing a pivotal pathogenic role in the syndrome.^21^ Collectively, these findings suggest that dysregulation of GnRH neuronal activity may underlie at least part of the neuroendocrine dysfunction associated with specific PMOS phenotypes. Consistent with this hypothesis, several studies using preclinical animal models of PMOS have highlighted the contribution of the brain to the neuroendocrine abnormalities associated with the syndrome^21^, ^22^, ^23^, ^24^, ^25^, ^26^, ^27^, ^28^, ^29^, ^30^, ^31^, ^32^, ^33^, ^34^, ^35^, ^36^, ^37^, ^38^ and suggested that chronically increased GnRH neuronal activity may represent a primary driver of reproductive dysfunction in PMOS.

However, GnRH receptor (GnRHR) antagonists have not yet been systematically evaluated as potential therapeutic approaches to correct the neuroendocrine and reproductive disturbances associated with the syndrome. An initial proof-of-concept in this direction was provided by Tata et al.,^29^ who demonstrated that short-term administration of the GnRHR antagonist Cetrorelix in a PMOS-like mouse model restored reproductive cyclicity and normalised androgen levels. These findings established proof-of-concept that attenuation of GnRHR signalling may represent a viable therapeutic strategy for PMOS.

This proof-of-concept study subsequently raised the question of whether alternative GnRHR antagonists with distinct pharmacokinetic properties might be suited for prolonged low-dose modulation of the reproductive axis. In this context, we focused on Ganirelix, a GnRHR antagonist whose shorter half-life and pharmacological profile may provide greater flexibility for achieving partial and reversible attenuation of LH hypersecretion without inducing profound suppression of reproductive function.

Ganirelix and Cetrorelix are synthetic decapeptide GnRH analogues with two d-amino acid substitutions at positions 1 and 6. The substitution in the amino-terminal domain at position 1 ablates receptor activation, while the substitution at position 6 enhances molecular folding, increases binding affinity, and decreases degradation.^39^ Both molecules act as competitive GnRHR antagonists with fully reversible effects upon discontinuation, but they differ in specific side-chain substitutions at position 6 that influence stability and receptor affinity.^39^ As a consequence, although structurally closely related, the two compounds differ in pharmacokinetic and pharmaceutical properties that become particularly relevant when investigating prolonged low-dose administration. Compared with Cetrorelix, Ganirelix exhibits a substantially shorter terminal half-life following a single 0.25 mg subcutaneous injection (approximately 13 h versus 30 h),^40^^,^^41^ thereby allowing greater flexibility and tighter control over the degree of pituitary suppression. This shorter pharmacokinetic profile is especially advantageous in exploratory dose-finding studies designed to achieve partial attenuation of LH pulsatility while minimising the risk of excessive or prolonged suppression of the reproductive axis.

These characteristics made Ganirelix particularly well suited for the present translational strategy, in which the objective was not to abolish gonadotrophin secretion, as in assisted reproductive technologies, but rather to fine-tune excessive GnRH/LH drive. Indeed, both Cetrorelix and Ganirelix are routinely used in *in vitro* fertilisation (IVF) procedures to prevent premature LH surges during controlled ovarian stimulation. Cetrorelix is administered either as a single 3 mg depot dose or as repeated 0.25 mg daily injections, whereas Ganirelix is used as a 0.25 mg daily injection, typically beginning on stimulation day 5 or 6 and continued until ovulation triggering. These regimens are intentionally designed to produce robust pituitary suppression and prevent premature ovulation but they would be unsuitable outside the IVF setting because they would inhibit ovulation and induce profound hypoestrogenism. By contrast, we hypothesised that carefully titrated subtherapeutic doses could selectively reduce pathological LH pulse frequency without fully shutting down the hypothalamic-pituitary-gonadal axis.

Given that accelerated LH pulsatility is considered a major driver of ovarian androgen excess in PMOS,^42^ and that experimental enhancement of LH secretion can induce PMOS-like traits in animal models,^23^^,^^28^^,^^43^ we reasoned that partial GnRHR antagonism might normalise neuroendocrine dynamics sufficiently to reduce androgen production while preserving reproductive function.

In the present study, we first performed a preclinical investigation to demonstrate that prolonged administration of low-dose Ganirelix restores oestrous cyclicity, ovulation, and physiological LH and testosterone levels in PMOS-like mice. To evaluate translational feasibility, we subsequently conducted a dose-finding pilot clinical study examining acute low-dose Ganirelix administration in women with PMOS. We evaluated two subtherapeutic doses and found that partial GnRH receptor antagonism reduces LH pulse frequency and circulating androgen levels, thereby mirroring the neuroendocrine improvements observed in our animal model. Collectively, these findings provide important evidence that low-dose GnRH antagonism may represent a promising therapeutic strategy for PMOS and establish a foundation for future phase-2 long-term clinical trials.

## Methods

### Animals and PMOS model induction

C57BL/6 J mice (Charles River, France; RRID: IMSR_JAX:000664) were housed at 21–22 °C with a 12 h light/dark cycle and provided ad libitum access to water and standard laboratory chow (9.5 mm Pelleted RM3, Special Diets Services; Competence Centre for Lab Animal Science of SAFE®; France). Mice were randomly assigned to groups of six animals per cage at the time of purchase or weaning with litters within the same treatment allocation group mixed (pseudo-random mixing based on weight) to minimise any potential bias. PAMH mice were generated according to previous studies.^29^ Pregnant adult (3–4 months) female mice were injected daily intraperitoneally (i.p.) at embryonic day (E) 16.5, 17.5, and 18.5 with either 200 μL of 0.01 M phosphate-buffered saline (PBS) only (control group) or 0.12 mg/kg/day human recombinant AMH_C_ (R&D Systems, Cat #: rhMIS 1737-MS-10) diluted in PBS (PAMH group). Mouse pregnancy was timed considering the detection of vaginal plugs as GD 0.5.

One group of animals (acute treatment; 8 weeks-old) was injected once intraperitoneally (i.p.) with 100 μL of a solution containing 0.01 M PBS (pH 7.4) with Ganirelix acetate (Sigma–Aldrich, Cat #: SML0241) at 0.5 mg/kg or with vehicle (0.01 M PBS). Another group of animals (long-term treatment; 8 weeks-old) was injected i.p. with 100 μL of a solution containing 0.01 M PBS (pH 7.4) with Ganirelix at 0.5 mg/kg or with vehicle (0.01 M PBS) three times a week for 6 weeks, with injections administered at two 2-day intervals and one 3-day interval per week. No animals were excluded from analyses.

### Ethics

Animal studies were approved by the Institutional Ethics Committees of Care and Use of Experimental Animals of the Universities of Lille 2 (France). All experiments were performed in accordance with the guidelines for animal use specified by the European Union Council Directive of September 22, 2010 (2010/63/EU) and were conducted in accordance with the ARRIVE (Animal Research: Reporting of in vivo Experiments) guidelines and the approved protocol (APAFIS# 29172-2020121811279767 v5) by the Ethical Committee of the French Ministry of Education and Research. All efforts were made to minimise animal suffering and animal care was supervised by veterinarians and animal technicians skilled in rodent healthcare and housing.

### Assessment of reproductive status and blood sampling in mice

Reproductive cycles were followed and characterised according to well-described guidelines of the mouse oestrous cycle according to well-established guidelines.^44^ Briefly, vaginal smears were collected using 10 μL of sterile saline, transferred to glass slides, and evaluated during the morning period at 8:00–9:00 a.m (room lights were on at 7:00 a.m). For the long-term preclinical study, vaginal smears were taken daily for 13 consecutive days before the Ganirelix treatment period to ascertain the phenotype of the mice and then during the last two weeks of the treatment (PBS or Ganirelix). Vaginal cytology was performed with freshly collected samples using an inverted microscope with maximum microscope condenser distance allowing a better contrast for proper visualisation. A cycle was considered complete if the sequence of proestrus followed by oestrus and then dioestrus was observed.

Tail-blood sampling for LH levels was conducted during the first day of dioestrus. For LH-level profile studies, mice were trained to interact with the investigator and allow restraint of the tail for at least two weeks before the beginning of each set of experiments. For the acute-treatment experiments, 4 μL of tail blood was taken every 5 min for 1 h (T_0_-T_60_), before the injection of PBS or Ganirelix, and then every 5 min for 2 h (T_60_-T_180_). For the long-term experiments, LH pulsatility was assessed at the end of the Ganirelix or PBS treatment, during the first dioestrus stage.

Samples were collected into Eppendorf tubes pre-loaded with 50 μL of 0.1 M phosphate-buffered saline (PBS)-0.05% Tween (pH = 7.4), thoroughly mixed, and snap-frozen with dry ice and stored at −80 °C. For sex-steroids measurements, blood was collected from the cheek at a single point for each treatment. Samples were kept in ice for 1 h followed by centrifugation at 6.8 rcf for 15 min at 4 °C. Plasma supernatant was transferred to sterile 1.5 mL Eppendorf tubes and stored at −80 °C.

### Hormone measurements and analysis in mice

For this study, we used an ultrasensitive ELISA method to measure circulating LH levels in the whole blood in mice as validated previously to assess pulsatile LH release in female mice over the oestrous cycle.^45^^,^^46^ Briefly, this ELISA uses a 96-well high-affinity binding microplate (Corning Costar® Cat #: 3590) coated with 50 μL of primary capture antibody (bovine LHβ subunit, Cat #: 518B7, RRID: AB_2665514; L. Sibley; University of California, UC Davis) at a dilution of 1:1000. The mouse LH-RP reference used for this assay was provided by Dr. Albert F. Parlow (National Hormone and Pituitary Program, Torrance, California, USA) and used to generate a standard curve with a twofold serial dilution from a start standard of 4 ng/mL to 0.0019 ng/mL LH-RP reference diluted in 0.2% bovine serum albumin (BSA; Sigma–Aldrich, Cat #: A9418)-0.1 M PBS-0.05% Tween solution. Blood samples were transferred to the coated as singlets for each point of sampling and incubated for 2 h under agitation at room temperature. A rabbit LH antiserum primary antibody (AFP240580Rb; NIDDK-NHPP; RRID: AB_2665533) was used at 1:10000 dilution and a secondary horseradish peroxidase-conjugated antibody (Streptavidin, Thermo-Fisher, Cat #: N200) was used at 1:8000 dilution. We used 100 μL of 1-Step Ultra TMB-Elisa Substrate Solution (ThermoFisher Scientific, Cat #: 34028) for the final revelation step followed by a stop solution with 50 μL of 3 M HCl per well. The assay sensitivity of this LH ELISA was 0.04 ng/mL and intra-assay coefficient of variation was 3.9% and the inter-assay coefficient of variation was 8.3%. LH pulse analysis was analysed based on previous studies.^28^^,^^46^ Briefly, mean LH levels were considered as the average of all measured values within the 3-h blood sampling protocol. LH pulse peaks were determined as a single point with a value more than 20% from 1 of the 2 previous points followed by a decrease by >10% in 1 of the next 2 subsequent points.^33^ Next, LH pulse frequency was determined by counting the number of identified pulses per hour.

The basal LH corresponds to the mean of the five lowest LH values over the study period before and after the treatment. Total LH is calculated as the sum of the LH values from T0 to T60 and from T60 to T180, divided by 2 (total LH per hour).

### Measurement of mouse steroid levels in serum by GC–MS/MS

Testosterone (T), Dihydrotestosterone (DHT), Oestradiol (E2), D4-androstenedione (4-dione), and Progesterone (Prog) were measured by GC–MS/MS. Twice charcoal dextran-stripped aged female rabbit serum was used as matrix for calibrators and quality control (QC) standards.

Briefly, each sample (200 μL of serum per mouse, calibration standards, quality controls, and blank matrix) was collected in a 4 mL borosilicate tube. A spiking solution of deuterated steroid internal standards (IS) (10 μL containing 0.5 ng of T-d_5_, 0.5 ng of DHT-d_3_, 50 pg of E2-d_4_, 2 ng of 4-dione-d_7_, and 3 ng of Prog-d_9_, except for blank matrix) (CDN Isotopes, Inc., Point-Claire, Canada), 200 μL of saline water, and 2.8 mL of 1-chlorobutane were added to each sample. After a 2 min vortex-step followed by rapid centrifugation, the upper organic phase was collected on a conditioned Hypersep SI 500 mg SPE mini-column (Thermo Scientific; Cat #: 60108-302). The column and adsorbed material were then washed with ethyl acetate/hexane (6 mL; 1/9). The second fraction containing the steroids of interest was eluted with ethyl acetate/hexane (4 mL; 1/1), then evaporated at 60 °C to dryness.

### Derivatization reactions and determination of steroid levels in the supernatant

T, DHT, and E2 were derivatised as previously described^47^ with pentafluorobenzoyl chloride (PFBC) (Sigma–Aldrich, Cat #: 103772-1G). Final extracts were reconstituted in 20 μL of isooctane, then transferred into conical vials for GC injection.

4-dione, and Progesterone were derivatised with 50 μL of heptafluorobutyric anhydride (Sigma–Aldrich, Cat #: H0396) and anhydrous acetone (1/1) mixture. Final extracts were reconstituted in 20 μL of anhydrous n-hexane, then transferred in conical vials for injection into the GC system (GC-2010 Plus, Shimadzu Corporation, Kyoto, Japan) using a 50% phenylmethylpolysiloxane VF-17MS capillary column (Agilent Technologies). A TQ8050 triple quadrupole mass spectrometer (Shimadzu Corporation, Kyoto, Japan) equipped with a chemical ionisation source (NCI), and operating in Q3 single ion monitoring (SIM) mode, was used for the detection of DHT, T, and E2, and with an electron impact source (EI) operating in multiple reaction monitoring (MRM) mode for the detection of 4-dione, and Progesterone. For NCI detection, the reagent gas was methane, and the GC was performed in pulsed splitless mode with a 1 min pulsed splitless-time. The oven temperature was initially 150 °C for 0.50 min, further increased to 305 °C at 20 °C/min and held at 305 °C for 3.60 min, and then to 335 °C at 30 °C/min and held at 335 °C for 1.7 min. The injection port and transfer line temperatures were respectively 290 and 280 °C. The flow rate of helium (carrier gas) was maintained constant at 0.96 mL/min. The mass spectrometer CI source temperature was 220 °C. For EI detection, GC was performed in the splitless mode with a 1-min splitless-time. The temperature in the oven was initially 70 °C for 1 min, further increased to 238 °C at 25 °C/min, and then to 261 °C at 5 °C/min. The injection port and transfer line temperatures were respectively 290 and 280 °C. The flow rate of carrier gas was maintained constant at 0.70 mL/min. The mass spectrometer EI source was 230 °C. The linearity of steroid measurement was confirmed by plotting the ratio of the steroid peak response/internal standard (IS) peak response to the concentration of steroid for each calibration standard. Accuracy, target ions, corresponding deuterated internal control, range of detection, low limit of quantification (LLOQ), and intra & inter assay CVs of the quality control are reported in Table 1.

**Table 1:** Analytical validation parameter of mouse steroid levels in serum by GC–MS/MS.

Accuracy (%)	Analytes	Target or Precursor ion analyte/IS∗ (*m*/*z*)	Range (pg)	Mean (pg/mL) *Intra- & Inter-assay CVs (%)*			
				LLOQ Mean Intra- & Inter-assay CVs	Low QC Mean Intra- & Inter-assay CVs	Middle QC Mean Intra- & Inter-assay CVs	High QC Mean Intra- & Inter-assay CVs
95–108	T/T-d_5_	482/487∗	10–2430	9.7	153.7	310.4	621.5
				*14.1*–*18.6*	*3.7*–*6.6*	*3.6*–*6.9*	*3.8*–*6.4*
95–109	DHT/DHT-d_3_	484/487∗	2–486	1.9	75.2	148.8	301.2
				*18.1*–*19.4*	*6.5*–*8.7*	*5.1*–*7.7*	*4.6*–*6.6*
94–107	E2/E2-d_4_	660/664∗	0.2–56	0.23	2.97	6.11	12.08
				*17.4*–*19.5*	*4.3*–*6.7*	*4.6*–*5.2*	*3.5*–*6.3*
93–110	4-dione/4-dione-d_7_	482.20 > 149.20 482.20 > 119.20 489.20 > 149.20∗ 489.20 > 119.20∗	10–2430	9.6	51.8	99.1	198.5
				*16.1*–*19.3*	*4.5*–*6.2*	*3.4*–*7.3*	*3.6*–*7.1*
95–107	Prog/Prog-d_9_	510.25 > 510.20 510.25 > 147.20 519.25 > 519.20∗ 519.25 > 147.20∗	20–4850	19.6	248.8	499.1	998.3
				*17.4*–*21.3*	*5.5*–*7.4*	*3.3*–*7.8*	*3.5*–*6.8*

Italics indicate intra- and inter-assay coefficients of variations (CVs), expressed as percentages.

∗ refer to the ions of deuterated steroids employed as internal standards.

### Mouse ovarian histology

Ovaries were collected from dioestrous mice at the end of the treatment (PBS or Ganirelix), immersion-fixed in 4% PFA solution and stored at 4 °C. Paraffin-embedded ovaries were sectioned at a thickness of 5 μm (histology core facility; PLBS UAR 2014 – US41) and stained with haematoxylin-eosin (Sigma Aldrich, Cat #: GHS132, HT1103128). Sections were examined throughout the ovary. Corpora lutea (CL) were classified and quantified as previously reported.^21^ To avoid repetitive counting, CL were counted every 100 μm by comparing the section with the preceding and following sections. CL were characterised by a central cavity filled with blood and follicular fluid remnants or by prominent polyhedral to round luteal cells.

### Physiological measurements

#### Body composition

Body composition was measured using a Minispec LF Series (Bruker Corporation). Fat and lean mass data are expressed as percentage body weight.

#### Glucose tolerance

Food was removed at 9 am, and after 5-h fasting, tail blood samples for insulin measurement were taken using glass capillaries before and 15 and 30 min after glucose administration. Blood samples were kept on ice before centrifugation (4 °C, 6.8 rcf, 15 min) and plasma was frozen at −80 °C until an ultrasensitive mouse insulin ELISA (Mercodia, Cat #: 10-1247-01) was performed on 12.5 μL of plasma per time point. Basal blood glucose levels were measured before i.p. glucose administration (2 g glucose kg^−1^ body weight) and 15, 30, 45, 60, 90 and 120 min after glucose administration using a Accu-Chek Guide glucometer (Roche Diabetes Care GmbH).

#### Insulin response measurement

Basal blood glucose levels were measured after a 5-h fast for the ITT before i.p. insulin administration (0.5 UI kg^−1^ body weight, Eli Lilly and Company, Cat #: HI0210) and 15, 30, 45, 60, 90, and 120 min after glucose administration using a Accu-Chek Guide glucometer (Roche Diabetes Care GmbH).

### Human trial design

A single-centre, parallel-group, phase I exploratory clinical study was conducted to evaluate the effects of low-dose Ganirelix on LH pulsatility in women with PMOS at the Lille University Hospital (CHU de Lille), France. Participants were recruited during endocrine gynaecology consultations at the Jeanne de Flandre Hospital, CHU Lille.

The primary objective was to determine whether low-dose GnRH receptor antagonism could reduce LH pulse frequency toward values observed in healthy women (target reduction approximately 20–30%). The study was conducted between April 2024 and February 2025 at the Jeanne de Flandre Hospital, CHU Lille.

Twenty women diagnosed with PMOS (all presenting phenotype A) were enrolled and admitted for a single-day hospital visit between day 2 (D2) and day 5 (D5) of either a spontaneous menstrual cycle or a dydrogesterone-induced withdrawal bleed. In women with infrequent spontaneous menses (n = 10), dydrogesterone was administered at a dose of 10 mg/day for 7 days and discontinued before study procedures to induce withdrawal bleeding and facilitate scheduling during the early follicular phase. This approach was used to minimise the likelihood of hormonal assessments being performed during the luteal phase, which could influence GnRH/LH pulsatility because of progesterone exposure.

Although spontaneous menstruation and progestin-induced withdrawal bleeding are not biologically identical, available evidence suggests that short-term dydrogesterone exposure does not significantly alter gonadotrophin secretion after treatment discontinuation.^48^^,^^49^ Hormonal assessments performed on D2-D5 following withdrawal bleeding were therefore considered appropriate for evaluation of LH pulsatility and steroid hormone concentrations.

An intravenous cannula was inserted, and blood samples were collected by a nurse at 10-min intervals during a 4-h baseline period (0–240 min) beginning at 08:00 h, followed by a second 4-h sampling period (240–480 min) after administration of Ganirelix (Orgalutran®, Organon France). Ganirelix was diluted in sterile water to a final volume of 1 mL and administered as a single subcutaneous injection at 240 min.

Participants were enrolled sequentially into two open-label dose cohorts. The first cohort (n = 10) received Ganirelix at a dose of 0.025 mg, and the second cohort (n = 10) received Ganirelix at a dose of 0.0625 mg. No randomisation procedures were used. Laboratory personnel and investigators performing hormonal and statistical analyses were blinded to dose allocation. The individual participant was the unit of assignment and analysis.

Serum testosterone, oestradiol, anti-Müllerian hormone (AMH), and follicle-stimulating hormone (FSH) concentrations were measured at baseline (T0) and at the end of the study period (T480). Participants were contacted by telephone 3–4 days after study completion to assess delayed adverse events. None were reported by the participants.

Two participants in the 0.025 mg cohort were excluded from the final analysis because hormonal profiles at enrolment were inconsistent with the early follicular phase, including oestradiol concentrations >300 pg/mL and baseline LH concentrations of 26 IU/L, suggestive of peri-ovulatory status.

#### Inclusion and exclusion criteria

Inclusion criteria: women aged 18–35 years, with a minimum weight of 51 kg, diagnosed with PMOS according to the Rotterdam criteria, consistent with current international guidelines,^9^ after exclusion of differential diagnoses, and with AMH levels >28 pmol/L, LH > 8 UI/mL and testosterone >0.39 ng/mL.

Eligible participants were required have a BMI lower than 30 kg/m^2^, to have discontinued all hormonal treatments or hormonal contraceptives for at least three months prior to enrolment and to be covered by the social security system.

Exclusion criteria: pregnancy, Metformin or other treatment affecting metabolism, inability to understand the study information sheet, or known hypersensitivity to Ganirelix, any structural analogue of gonadotrophin-releasing hormone (GnRH), exogenous peptide hormones, or formulation excipients.

#### Endpoints and outcome measures

The primary outcome was the change in the number of LH pulses during the 4-h post-Ganirelix phase compared with the 4-h pre-treatment phase.

Secondary endpoints:

Secondary outcomes included changes in mean, total, and basal LH concentrations, before and after Ganirelix administration. Basal LH was defined as the mean of the five lowest LH values within each phase.

Additional secondary outcomes included changes in testosterone, D4-androstenedione, FSH, oestradiol, and AMH levels between baseline (T0) and the end of the study period (T480).

#### Post-treatment safety assessment

Adverse effects among women were assessed through a follow-up telephone interview performed on days 3–4 after treatment to identify any potential safety concerns or side effects. No safety issues were detected. One participant reported a mild headache that resolved spontaneously, and the association with Ganirelix could not be confirmed.

### Ethics

Ethical approval for the clinical study conducted in women with PMOS was obtained from the Agence Nationale de sécurité du medicament et des produits de santé (ANSM; N° EudraCT: 2023-000176-35) and an independent ethics committee (Comité de Protection des Personnes Ile de France VI, number 2023-000176-35). The clinical protocol was registered on ClinicalTrials.gov (ID: NCT05751252). The study was conducted in accordance with the Declaration of Helsinki and International Council for Harmonisation guidelines on Good Clinical Practice. The patients received complete information about the study and provided written informed consent before undergoing study-specific procedures.

### Hormones’ measurements in women with PMOS

LH concentrations in blood samples collected over the 8-h period were measured by an automated chemiluminescent immunoassay on an Architect i2000 SR analyser (Abbott Lab, Abbott Park, USA) as previously described.^50^

Serum FSH, Total Testosterone (TT) and Oestradiol levels were measured by an automated chemiluminescent immunoassay on an Architect analyser (Abbott Lab, USA). Serum D4-androstenedione (4-dione) was measured by LC-MS/MS.

AMH was measured by a fully automated sandwich chemiluminescent immunoassay using the Unicel DxI 800 Access Immunoassay system (Beckman Coulter Inc, Brea, CA, USA) as previously described.^51^

For steroids, the limit of quantification of the assay was 20 pg/mL for oestradiol, 0.02 ng/mL for TT and 0.012 ng/mL for 4-dione. Reference ranges in young women in follicular phase were as follows: LH, 2.0–7.6 UI/L; FSH, 4.3–9.0 UI/L^52^; TT, 0.13–0.53 ng/mL^53^; Oestradiol, 21–251 pg/mL; 4-dione, 0.52–1.98 ng/mL^54^; AMH, 11–30.3 pmol/L.^55^

### LH pulsatile assessment in women with PMOS

The same analytical method, previously described in the literature, was applied to determine LH pulses in both women and mice, despite differences in study duration and sampling frequency.^33^ To be considered an LH pulse or peak, the value had to show an increase of more than 20% compared with one of the two preceding points, followed by a decrease of more than 10% in one of the two subsequent points. The mean LH is the average of all LH values over the study period. The basal LH corresponds to the mean of the five lowest LH values over the study period. The total LH corresponds to the sum of all LH values at T0-T240 and T240-T480.

### Statistics and data analysis

All analyses were performed using 10.0.2 GraphPad Prism software (GraphPad; San Diego, CA, USA; RRID:SCR_002798). The normality of each group was determined by Shapiro–Wilk test. We did not assume equal variances. Statistically significant *P* values were considered when *P* < 0.05. No statistical methods were used to pre-determine sample sizes but our sample sizes were similar to those reported in previous publications for both the preclinical and clinical investigations.^56^, ^57^, ^58^ Animals were randomised to experimental groups and investigators were blinded to group allocation during data collection and analyses. We did not exclude animals from our experimental groups. There were no exclusions of experimental units or data points.

For the clinical trial, a sample size of 10 per study group is regarded typical of proof-of-concept studies and is considered adequate for the objectives of the study to be achieved. Indeed, our sample size of 10 per study group compares favourably with previous works examining the effects of other GnRH antagonists' administration on reproductive hormone secretion in both healthy volunteers and patients with PMOS.^17^^,^^59^ No interim analysis of the dataset was undertaken to guide the sample size. Recruitment was stopped when sufficient numbers had enrolled to ensure the planned sample size was achieved, and the study was stopped once all participants had completed the study protocol, as no safety concerns occurred requiring early termination. Participants were not randomised in the clinical study and they were enrolled sequentially into two open-label dose cohorts. The first cohort (n = 10) received Ganirelix at a dose of 0.025 mg, and the second cohort (n = 10) received Ganirelix at a dose of 0.0625 mg. The participants and the laboratory personnel performing the hormonal assays and statistical analyses were blinded to dose allocation. Within-group comparisons between pre-treatment and post-treatment phases were performed using paired statistical analyses. For the normal distribution, data were compared using paired two-sided Student's *t*-test (2 groups before and after the treatment) and two-way ANOVA (multiple groups) followed by Tukey's multiple comparisons tests. For non-normally distributed values, Mann–Whitney U test (2 groups) was used.

### Role of funders

The funding sources for this project played no role in the study design, data collection, analysis, interpretation, writing, or editing of the manuscript.

## Results

### Acute low-dose Ganirelix administration reduces LH dynamics in a PMOS-like mouse model

**Fig. 1:**
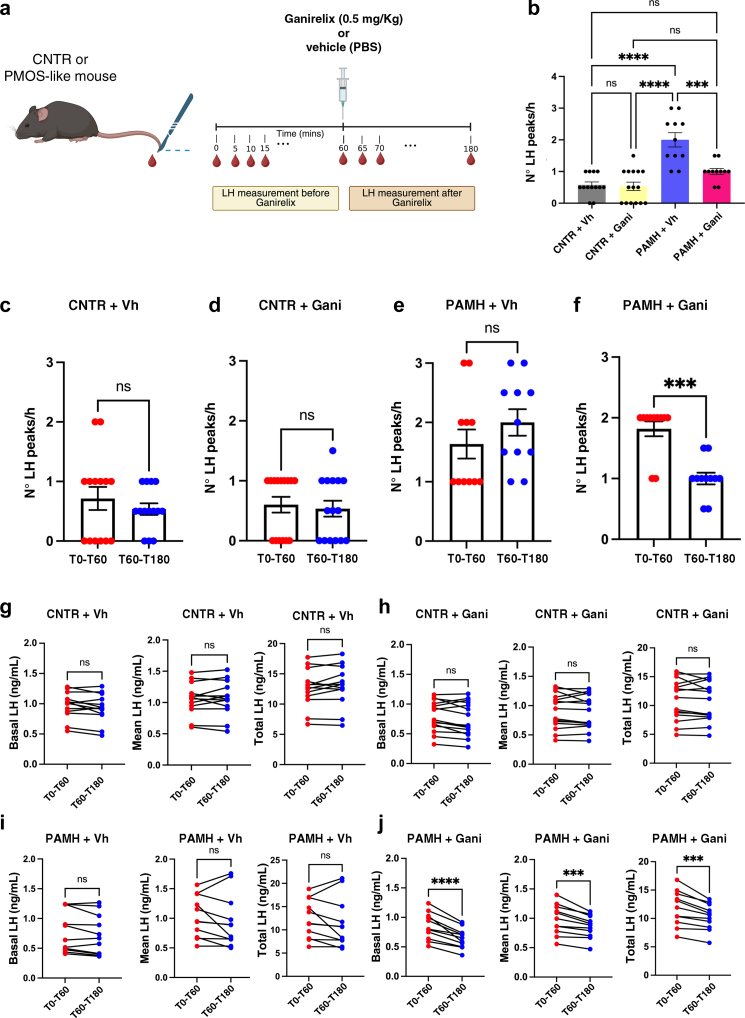
**Acute administration of low-dose Ganirelix restores normal LH pulsatility and LH secretion in PMOS-like mice without affecting controls.** (**a**) Schematic of the experimental design. Control (CNTR) and PMOS-like (PAMH) mice received a single intraperitoneal injection of Ganirelix (0.5 mg kg^−1^) or vehicle (PBS) at dioestrus. Blood samples were collected every 5 min during the first hour preceding the acute treatment (T_0_–T_60_) and during the 2 h following the injection of vehicle or Ganirelix (T_60_–T_180_). (**b**) The mean number of LH pulses per hour was calculated and analysed among the four groups (CNTR + vehicle: *n* = 13; CNTR + Ganirelix: *n* = 15; PAMH + vehicle: *n* = 11; PAMH + Ganirelix: *n* = 11). Data are presented as mean ± SEM. Statistical analysis by two-way ANOVA with Tukey's multiple comparisons test; ns = not significant, ∗∗∗*p* < 0.0005, ∗∗∗∗*p* < 0.0001. (**c–f**) Within-group comparisons of LH pulsatility in the four treatment groups. Data are presented as mean ± SEM. Data were analysed by paired two-tailed t-test; ns = not significant, ∗∗∗*p* < 0.001. In CNTR mice, neither vehicle (**g**) nor Ganirelix (**h**) affected basal, mean, or total LH levels. In PAMH mice, vehicle (**i**) had no effect, whereas Ganirelix (**j**) significantly reduced basal, mean, and total LH concentrations, indicating normalisation of exaggerated LH secretion. Each line represents an individual animal; red and blue symbols denote pre- and post-treatment values, respectively. In **g–j**, data were analysed by paired two-tailed t-test; ns = not significant, ∗∗∗p < 0.001, ∗∗∗∗p < 0.0001.

To assess the acute impact of GnRHR antagonism on LH secretion, control (CNTR) and PAMH adult mice (2–3 months-old) received a single low dose of Ganirelix (0.5 mg/kg; Fig. 1a). This dose was informed by previous work using the same dose of the related antagonist Cetrorelix in PMOS-like mice,^29^ where effects were only assessed after two weeks of treatment. In contrast, the present study investigated both the acute and long-term (6-week) effects of Ganirelix. To monitor LH dynamics, 4 μL tail-blood serial samples were collected every 5 min for 1 h prior to injection and for 2 h following administration of either vehicle (PBS) or Ganirelix (Fig. 1a).

As expected, based on our previous studies,^29^ PAMH mice treated with vehicle displayed elevated LH pulsatility levels compared with CNTR animals (Fig. 1b). Ganirelix treatment markedly reduced LH pulse frequency in PAMH mice but had no effect in CNTR animals.

Within-group comparisons confirmed the lack of significant changes in CNTR animals treated with either vehicle or Ganirelix (Fig. 1c and d) and no effect of vehicle injection in the PAMH group (Fig. 1e). On the other hand, Ganirelix significantly decreased LH pulse frequency in PAMH mice (Fig. 1f).

Further analyses showed that in CNTR mice, neither vehicle nor Ganirelix administration produced any significant changes in basal LH, mean LH, or total LH secretion (Fig. 1g and h), confirming that low-dose GnRHR antagonism does not disrupt physiological LH dynamics under normal conditions. In PAMH mice, a single PBS injection did not alter LH secretion dynamics (Fig. 1i). By contrast, Ganirelix administration led to a marked suppression of LH output, reflected by significant reductions in basal LH, mean LH, and total LH secretion compared with pre-treatment values (Fig. 1j). These findings indicate that low-dose GnRHR antagonism selectively normalises the pathological LH hyperpulsatility and secretion characteristic of the PMOS-like state, while leaving physiological LH secretion in control mice unaffected.

### Intermittent long-term Ganirelix treatment corrects hyperandrogenism in PMOS-like mice

After confirming that a low-dose Ganirelix acutely suppresses LH dynamics in PAMH female mice, we next examined the effects of long-term Ganirelix treatment.

**Fig. 2:**
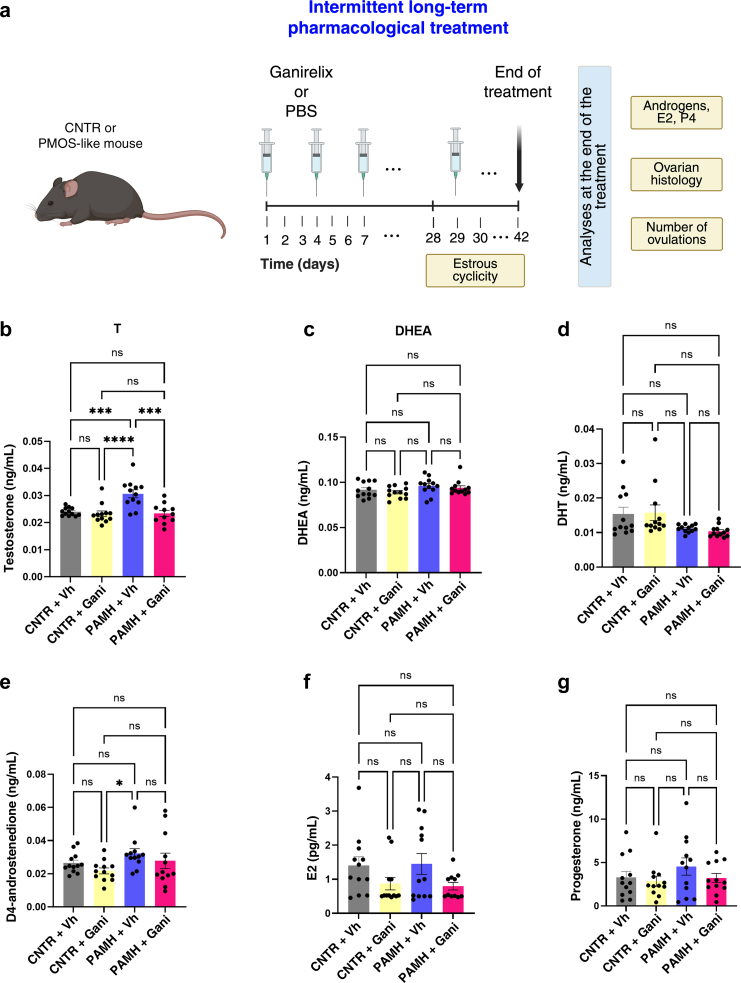
**Long-term low-dose Ganirelix treatment restores reproductive and hormonal balance in PMOS-like mice.** (**a**) Experimental design. Control (CNTR) and PMOS-like (PAMH) mice received intermittent subcutaneous injections of Ganirelix (0.5 mg kg^−1^) or vehicle (PBS) three times per week for six weeks. Oestrous cyclicity was monitored throughout treatment and analyses were performed at the study endpoint at first dioestrous stage in the four groups to assess mean circulating levels of testosterone (**b**; CNTR + Vh: *n* = 12; CNTR + Gani, *n* = 12; PAMH + Vh, *n* = 12; PAMH + Gani, *n* = 11), DHEA (**c;** CNTR + Vh: *n* = 12; CNTR + Gani, *n* = 12; PAMH + Vh, *n* = 12; PAMH + Gani, *n* = 12), DHT (**d**; CNTR + Vh: *n* = 12; CNTR + Gani, *n* = 12; PAMH + Vh, *n* = 12; PAMH + Gani, *n* = 12), D4-androstenedione (**e**; CNTR + Vh: *n* = 12; CNTR + Gani, *n* = 12; PAMH + Vh, *n* = 12; PAMH + Gani, *n* = 12), Oestradiol (E2, **f**; CNTR + Vh: *n* = 12; CNTR + Gani, *n* = 12; PAMH + Vh, *n* = 12; PAMH + Gani, *n* = 11) and progesterone (**g**; CNTR + Vh: *n* = 12; CNTR + Gani, *n* = 12; PAMH + Vh, *n* = 12; PAMH + Gani, *n* = 12). Each dot represents an individual mouse. Data are presented as mean ± SEM. Statistical analysis by two-way ANOVA with Tukey's multiple comparisons test; ns = not significant, ∗*p* < 0.05, ∗∗∗*p* < 0.0005, ∗∗∗∗*p* < 0.0001.

CNTR and PAMH mice (2 months old) were treated intermittently with Ganirelix or vehicle (PBS) three times per week for six weeks. Hormonal and reproductive parameters were assessed at the end of treatment (Fig. 2a). We used young adult female animals so that, by the end of the experiments, they would be 14 weeks-old, a time point at which PAMH animals exhibit the lean PMOS phenotype.^29^^,^^60^ Moreover, a previous study reported that the PAMH-induced PMOS model exhibited no detectable metabolic alterations at 12 weeks of age, including changes in body weight, adiposity, and glucose homoeostasis.^61^ Here, we confirmed the absence of confounding metabolic alterations at 16 weeks of age (Supplementary Figure S1). Specifically, body weight, body fat composition, glucose homoeostasis, and insulin levels were not significantly altered in PAMH mice at this age (Supplementary Figure S1a–d).

As expected, based on our previous studies,^29^ PAMH mice treated with vehicle displayed elevated testosterone levels compared with CNTR animals at the end of the treatment period (Fig. 2b). Long-term Ganirelix administration normalised testosterone concentrations, whereas these parameters remained unaffected in CNTR mice (Fig. 2b). In contrast, circulating levels of DHEA, DHT, D4-androstenedione, oestradiol (E_2_), and progesterone were not significantly altered by Ganirelix treatment in either group (Fig. 2c–g).

Together, these findings indicate that chronic, long-term, intermittent low-dose GnRHR antagonism specifically attenuates pathological hyperandrogenism in PMOS-like mice.

### Intermittent long-term Ganirelix treatment rectifies oestrous cyclicity and ovulation in PAMH mice

**Fig. 3:**
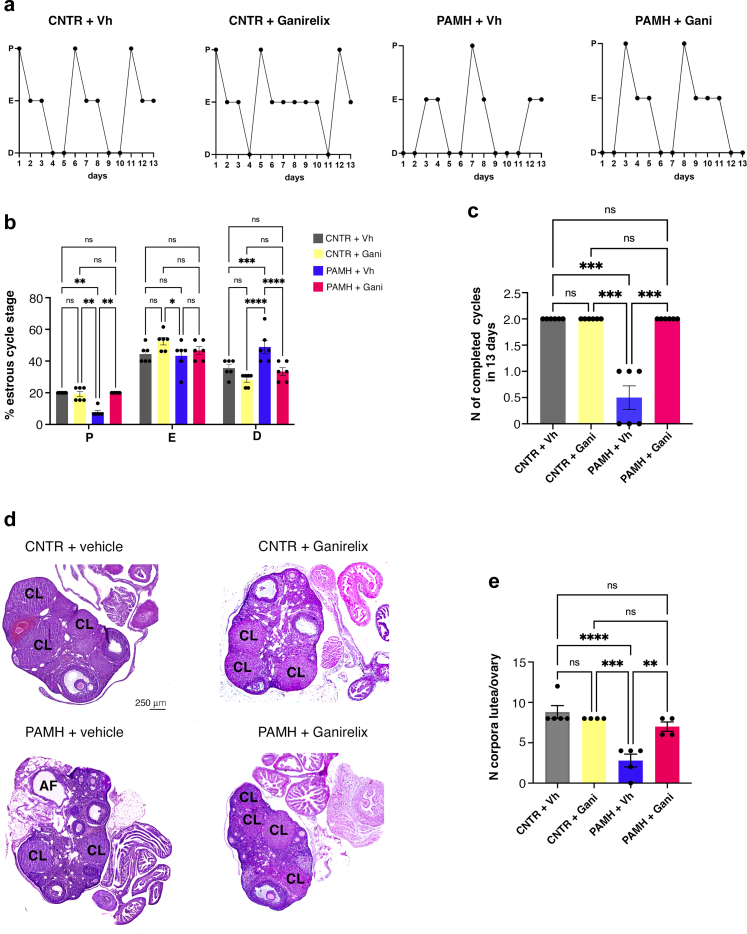
**Long-term Ganirelix treatment restores oestrous cyclicity and ovulation in PMOS-like mice.** (**a**) Representative oestrous cycle profiles over 13 consecutive days in control (CNTR) and PMOS-like (PAMH) mice treated with vehicle (Vh) or Ganirelix (Gani). P, proestrus; E, oestrus; D, dioestrus. (**b**) Percentage of time spent in each oestrous stage. PAMH mice exhibited prolonged dioestrus and disrupted cyclicity, which were normalised by Ganirelix treatment (CNTR + Vh, *n* = 6; CNTR + Gani, *n* = 6; PAMH + Vh, *n* = 6; PAMH + Gani, *n* = 6). (**c**) Number of completed oestrous cycles within 13 days (CNTR + Vh, *n* = 6; CNTR + Gani, *n* = 6; PAMH + Vh, *n* = 6; PAMH + Gani, *n* = 6). (**d**) Representative ovarian histology (H&E staining) showing corpora lutea (CL) and antral follicles (AF). PAMH mice treated with Ganirelix displayed multiple CL, indicating restored ovulation. Scale bar = 250 μm. (**e**) Quantification of corpora lutea per ovary confirming increased ovulatory activity in PAMH mice following Ganirelix treatment (CNTR + Vh, *n* = 5; CNTR + Gani, *n* = 4; PAMH + Vh, *n* = 5; PAMH + Gani, *n* = 4). Data are presented as mean ± SEM; each dot represents an individual mouse. Statistical significance determined by two-way ANOVA with Tukey's multiple comparisons test ns = not significant, ∗∗*p* < 0.01, ∗∗∗*p* < 0.001, ∗∗∗∗*p* < 0.0001.

To evaluate reproductive outcomes, oestrous cycles were monitored, during the last two weeks of the treatment, in CNTR and PAMH mice. CNTR animals, regardless of treatment, displayed regular cycles with normal transitions through proestrus, oestrus, and dioestrus (Fig. 3a–c). In contrast, PAMH mice treated with PBS exhibited disrupted cycles, with prolonged dioestrus and significantly fewer completed cycles (Fig. 3a–c). Strikingly, Ganirelix treatment restored cyclicity in PAMH mice, normalising the distribution of cycle stages and increasing the number of completed cycles to levels comparable to CNTR controls (Fig. 3b and c).

Histological analyses further revealed that ovaries from vehicle-treated PAMH mice displayed atretic follicles and a reduced number of corpora lutea, consistent with anovulation (Fig. 3d). By contrast, Ganirelix treatment markedly increased the number of corpora lutea in PAMH mice, indicating rescue of ovulatory function (Fig. 3d and e). As expected, CNTR ovaries exhibited abundant corpora lutea regardless of treatment.

Together, these findings demonstrate that intermittent long-term low-dose GnRHR antagonism restores oestrous cyclicity and ovulation in PMOS-like mice, without disrupting normal reproductive function in controls.

### Acute effects of low-dose Ganirelix on LH pulsatility in women with PMOS

Our preclinical findings suggest that intermittent long-term GnRHR antagonism could improve neuroendocrine and reproductive outcomes in women with PMOS. However, before initiating such long-term trials, it is critical to identify the optimal dose of a GnRHR antagonist that reliably normalises LH pulsatility in women with PMOS, as this is the prerequisite for reducing androgen excess and reinstating ovulation. This rationale motivated us to conduct a clinical trial to define the dose–response relationship of low-dose Ganirelix in women with PMOS.

We conducted a phase I, single-centre pilot study with two parallel groups to evaluate the efficacy of two different doses of Ganirelix in reducing aberrant LH secretion and steroid hormones in women with PMOS. To minimise the confounding effect of insulin resistance, which exacerbates hyperandrogenism in women with PMOS, only patients without obesity (BMI <30) were included in this study that was performed from April 2024 to February 2025 at the CHU of Lille, France.

Twenty women were initially recruited to receive Ganirelix at either 0.025 mg (*n* = 10) or 0.0625 mg (*n* = 10), corresponding to one-tenth and one-fourth of the standard clinical dose employed in IVF protocols. However, two patients of the Ganirelix arm at 0.025 mg were excluded from the analysis because their baseline hormone profiles were not consistent with the early follicular phase. Both presented with oestradiol levels >300 pg/mL and baseline LH concentrations of 26 IU/L, findings suggestive of the peri-ovulatory phase rather than the intended early follicular phase at enrolment.

The two groups were comparable in terms of age (24.6 ± 4.2 vs. 26.0 ± 3.6 years) and BMI (24.6 ± 3.9 vs. 24.0 ± 2.7 kg/m^2^; Table 2). The hormone values reported in Table 2 correspond to measurements obtained at T0, on the day of study initiation, and they are consistent with the PMOS phenotype during the early follicular phase (D2-D5).

**Table 2:** Baseline characteristics of women with PMOS included in the clinical trial.

	PMOS + Ganirelix 0.025 mg (n = 8)	PMOS + Ganirelix 0.0625 mg (n = 10)
Age (years)	24.6 ± 4.2	26.0 ± 3.6
BMI (kg/m^2^)	24.6 ± 3.9	24.0 ± 2.7
LH (IU/L)	6.9 ± 3.7	9.0 ± 5.1
FSH (IU/L)	5.4 ± 1.6	4.7 ± 1.4
LH/FSH	1.3 ± 0.5	1.9 ± 0.7
Oestradiol (pg/mL)	36.1 ± 9.9	36.0 ± 12.7
AMH (pmol/L)	71.3 ± 17.9	73.0 ± 25.9
Testosterone (ng/mL)	0.6 ± 0.2	0.5 ± 0.2
D4-androstendione (ng/mL)	2.4 ± 1.0	2.0 ± 0.8

Mean ± S.D. are presented. PMOS: polyendocrine metabolic ovarian syndrome; BMI: body mass index; LH: luteinising hormone; FSH: Follicle stimulating hormone; AMH: anti-Müllerian hormone. LH values indicated in Table 2 reflect concentrations measured at T0 on the day of the trial and are distinct from screening values used to determine eligibility. All participants met the inclusion criterion of LH > 8 IU/mL at the time of initial screening.

**Fig. 4:**
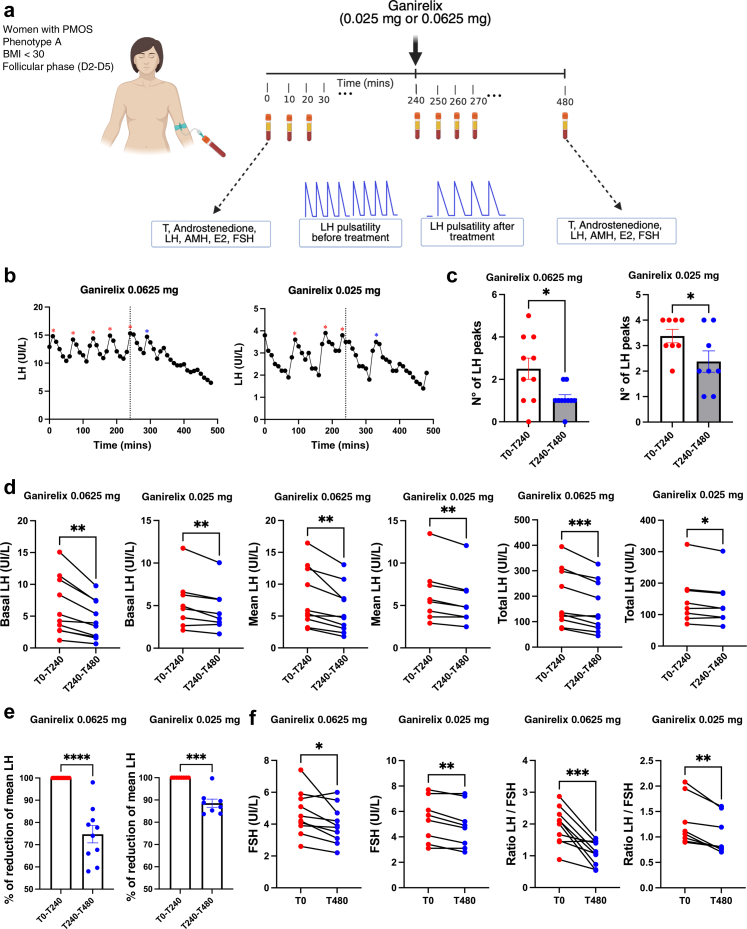
**Acute effects of low-dose Ganirelix on LH pulsatility and gonadotrophin secretion in women with PMOS.** (**a**) Experimental design. Women with PMOS received a single subcutaneous injection of Ganirelix (0.025 or 0.0625 mg) during the early follicular phase (D2–D5). Blood samples were collected every 10 min for 8 h to assess LH pulsatility and circulating hormone levels (T, androstenedione, LH, AMH, E2, and FSH) before and after treatment. (**b**) Representative LH pulse profiles before and after Ganirelix administration. (**c**) Number of LH pulses recorded before (T_0_–T_240_) and after (T_240_–T_480_) treatment (Ganirelix 0.0625 mg, *n* = 10; Ganirelix 0.025 mg, *n* = 8). Both doses significantly reduced LH pulse frequency. (**d**) Paired comparisons of basal, mean, and total LH secretion before and after treatment showing dose-dependent suppression of LH output (Ganirelix 0.0625 mg, *n* = 10; Ganirelix 0.025 mg, *n* = 8). (**e**) Percentage reduction of mean LH levels after Ganirelix administration (Ganirelix 0.0625 mg, *n* = 10; Ganirelix 0.025 mg, *n* = 8). (**f**) FSH levels and LH/FSH ratios before and after treatment. Ganirelix decreased LH/FSH ratios while preserving FSH secretion (Ganirelix 0.0625 mg, *n* = 10; Ganirelix 0.025 mg, *n* = 8). Each dot represents an individual participant; red and blue points denote pre- and post-treatment values, respectively. Data are presented as mean ± SEM. Statistical significance determined by paired two-tailed t-test; ∗*p* < 0.01, ∗∗*p* < 0.001, ∗∗∗*p* < 0.0001, except for **e** and **f** in which Mann–Whitney test (two-tailed) was applied, ∗∗∗*p* < 0.0005, ∗∗∗∗*p* < 0.0001.

To assess the short-term effects of GnRHR antagonism, women with PMOS received a single subcutaneous injection of Ganirelix (0.025 mg or 0.0625 mg) during the early follicular phase (D2-D5). To monitor LH dynamics, blood samples were collected every 10 min for 4 h prior to injection and for an additional 4 h after treatment (Fig. 4a).

Healthy women present approximately one LH pulse every 90–100 min throughout the early to mid-follicular phase (≈0.6–0.7 LH pulse/hour).^62^^,^^63^ In contrast, women with PMOS have an inherent abnormality in the GnRH system with higher pulse frequency than controls (∼0.9–1 pulse per hour).^10^^,^^12^^,^^16^

Representative time courses demonstrate that both doses of Ganirelix rapidly reduced the abnormal LH pulse frequency of women with PMOS (Fig. 4b). Quantitative analysis revealed a significant reduction in the number of LH pulses within 4 h of administration in both the 0.025 mg and 0.0625 mg groups, reaching respectively ∼29% and ∼62% reduction in LH pulse frequency as compared to the pre-treatment temporal window (Fig. 4c).

Basal, mean, and total LH concentrations were also significantly decreased at both doses (Fig. 4d), corresponding to a ∼12–27% reduction in mean LH secretion using the two doses respectively (Fig. 4e).

FSH concentrations were also significantly reduced following Ganirelix administration levels (Fig. 4f), although to a much lesser extent than LH. Finally, both doses of Ganirelix significantly decreased the LH/FSH ratio (Fig. 4f).

These findings demonstrate that acute low-dose GnRHR antagonism tempers LH hypersecretion and the LH/FSH ratio in women with PMOS, closely mirroring the effects observed in the preclinical mouse model.

### Acute effects of Ganirelix on steroid hormones and AMH in women with PMOS

To investigate whether acute suppression of LH by Ganirelix impacts downstream steroidogenesis, circulating sex steroids and AMH were measured at baseline (T0) and at the end of the treatment (T480).

**Fig. 5:**
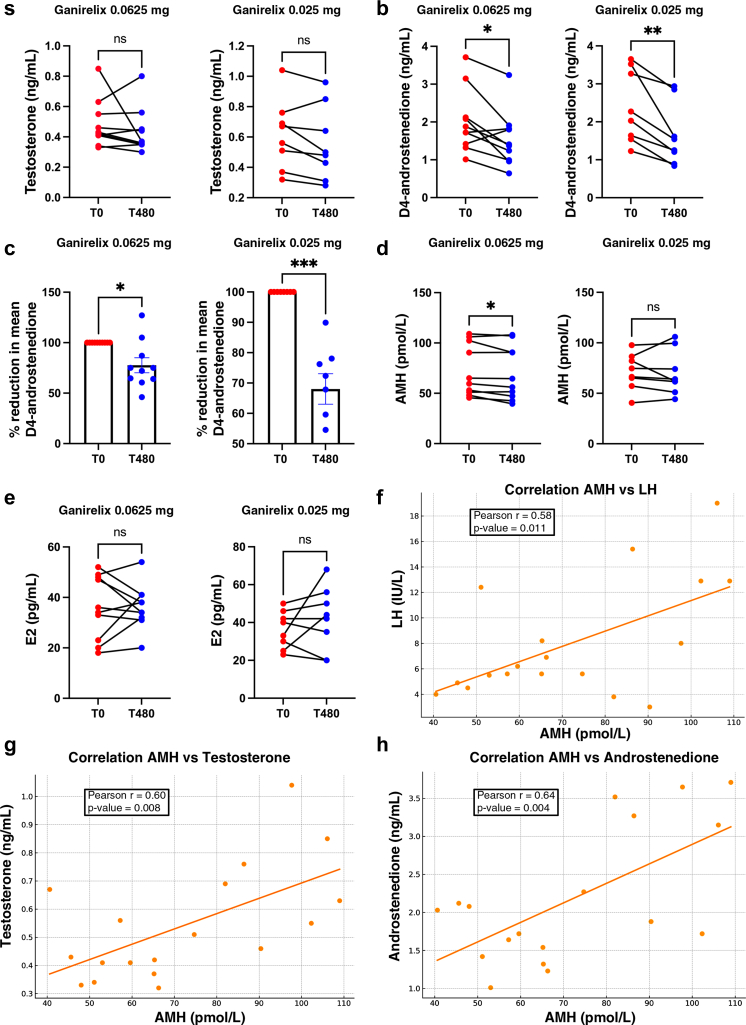
**Low-dose Ganirelix reduces circulating androgens and modulates AMH levels in women with PMOS.** (**a**) Serum testosterone and (**b**) D4-androstenedione concentrations before (T_0_) and 8 h after (T_480_) administration of Ganirelix (0.025, *n* = 8 or 0.0625 mg, *n* = 10). Both doses significantly reduced D4-androstenedione, while testosterone levels remained unchanged. (**c**) Percentage reduction in mean D4-androstenedione concentrations following treatment. (**d**) Circulating anti-Müllerian hormone (AMH) levels before and after Ganirelix injection. AMH decreased significantly with the 0.0625 mg dose. (**e**) Oestradiol (E2) levels remained stable across both doses, indicating preservation of normal oestrogenic milieu. (**f**) Correlation analyses showing positive associations between AMH and LH, testosterone (**g**), and D4-androstenedione (**h**) levels in women with PMOS (Pearson correlation). Each dot represents an individual participant; red and blue points indicate pre- and post-treatment values, respectively (Ganirelix 0.025, *n* = 8; Ganirelix 0.0625 mg, *n* = 10). Data are presented as mean ± SEM. Paired two-tailed t-test was used for analysis in **a**, **b**, **d** and **e**; ns = not significant, *p* > 0.05, ∗*p* < 0.01, ∗∗*p* < 0.001. Mann–Whitney test two tailed was applied in **c**, ∗∗∗*p* < 0.0005, ∗*p* < 0.05.

Testosterone concentrations remained unchanged at both doses (Fig. 5a). In contrast, D4-androstenedione levels were significantly reduced following Ganirelix administration, both at 0.0625 mg and 0.025 mg, corresponding to a mean reduction of ∼25–30% (Fig. 5b and c). AMH levels also declined modestly but significantly in the 0.0625 mg group, while no change was observed in the 0.025 mg group (Fig. 5d). Oestradiol concentrations were unaffected at either dose (Fig. 5e).

Correlation analyses revealed that baseline AMH levels were positively associated with circulating LH (*r* = 0.58, *p* = 0.011), testosterone (*r* = 0.60, *p* = 0.008), and D4-androstenedione (*r* = 0.64, *p* = 0.004) (Fig. 5f–h).

Taken together, these results demonstrate that acute low-dose GnRHR antagonism in women with PMOS effectively reduces LH pulsatility and normalises the LH/FSH ratio, while concomitantly lowering circulating androgens.

## Discussion

PMOS is a common condition associated with reproductive and metabolic complications. One of the hallmarks of its complex pathophysiology is neuroendocrine dysfunction, characterised by aberrant GnRH secretion.^4^^,^^64^ Consistent with this, previous studies have shown that chronic overactivation of GnRH neuronal network in otherwise healthy adult mice are sufficient to recapitulate PMOS-like reproductive and endocrine traits, including lasting neuroendocrine dysfunction, irregular reproductive cycles, elevated androgen levels, and ovarian abnormalities.^23^^,^^28^ These data indicate that a primary central disturbance in the GnRH system can independently trigger a self-perpetuating cascade of neuroendocrine and reproductive disruptions resembling adult PMOS pathology.

Previous studies from our group using the GnRHR antagonist Cetrorelix in PMOS-like mouse models were limited to short-term treatment durations of approximately two weeks,^29^ leaving the long-term consequences of GnRHR antagonism largely uncharacterised. In the present study, we evaluated Ganirelix, an antagonist with a distinct molecular structure and pharmacokinetic profile, in both acute and extended treatment contexts, assessing its immediate effects on LH secretion dynamics and its sustained impact over a six-week period on reproductive cyclicity, ovulation, and androgen production. A key finding of this study was that a single low dose of Ganirelix acutely normalised exaggerated LH secretion in PAMH mice while preserving physiological LH output in control mice.

This selectivity is consistent with the competitive pharmacodynamics of GnRHR antagonism^65^, ^66^, ^67^: under physiological conditions, normal GnRH pulse frequency and amplitude result in relatively low and intermittent receptor occupancy, rendering low-dose Ganirelix insufficient to meaningfully reduce net GnRHR activation.^40^^,^^68^ In contrast, the pathologically elevated GnRH pulsatility in PMOS may chronically increase receptor occupancy, selectively sensitising the system to partial competitive blockade at this dose.

This selectivity is important because complete LH suppression, as achieved with high-dose regimens in assisted reproductive technologies, prevents ovulation and would be counterproductive as a chronic therapy for PMOS.

A second important finding is that intermittent long-term administration of low-dose Ganirelix over six weeks effectively restored oestrous cyclicity, normalised circulating testosterone levels, and reinstated ovulation in PAMH mice. These results extend our previous foundational observations,^29^ showing that shorter Cetrorelix treatment of PAMH mice was sufficient to restore reproductive cyclicity and normalise androgen levels, establishing proof-of-concept that attenuation of GnRHR signalling may constitute a viable therapeutic strategy in PMOS. That two pharmacokinetically distinct GnRHR antagonists, administered at different durations, converge on comparable neuroendocrine and reproductive outcomes strongly suggests a class effect, reinforcing the notion that partial attenuation of GnRHR signalling, irrespective of the specific compound employed, is sufficient to interrupt the pathological neuroendocrine cascade driving PMOS-like traits.

It is important to note that our preclinical experiments were conducted in young adult, lean, metabolically healthy PMOS-like mice. Indeed, as we have shown in this study, PAMH mice at 4 months of age do not yet display overt metabolic alterations. This model is known to progressively develop features such as increased adiposity, insulin resistance, and glucose intolerance by approximately 6 months of age.^60^ Indeed, these metabolic disturbances emerge later than the reproductive and neuroendocrine abnormalities that were the focus of the present work.

Our findings extend the preclinical observations on the PAMH model to women with PMOS, demonstrating that administration of low-dose Ganirelix at 0.025 mg and 0.0625 mg, equivalent to one-tenth and one-fourth of the standard dose used in IVF protocols, rapidly reduced LH pulse frequency and normalised the LH-to-FSH ratio. Notably, both doses attenuated LH pulsatility without causing complete suppression, resulting in a 29% and 62% reduction in LH pulses/hour, as well as a 12% and 27% reduction in mean LH secretion, respectively. Importantly, both FSH levels and the LH/FSH ratio declined significantly following injection of either Ganirelix dose, consistent with its expected mechanism of action.

Both doses of Ganirelix also significantly lowered circulating D4-androstenedione by ∼25–30%, while testosterone levels remained unchanged 4 h after the injection of the antagonist. Hayes et al.^59^ showed that testosterone decreased within 4 h after injection of another GnRHR antagonist (Nal-Glu GnRHR antagonist), with nadir values reached only at 8 h. Given that testosterone is synthesised from androstenedione, the more rapid decline in D4-androstenedione observed in our study is consistent with its role as a precursor. Therefore, it is likely that testosterone levels had not yet reached their nadir by the final sampling point at T480, and that more pronounced decreases in testosterone concentrations might have been observed if measurements had been extended to the following day.

This differential response can also be explained by the distinct sources and regulatory mechanisms of these androgens. Circulating androstenedione is derived approximately equally from the ovary (50%) and the adrenal gland (50%), whereas circulating testosterone originates only about 30% from the ovary and roughly 70% from peripheral conversion of androstenedione and other precursors.^49^ GnRH antagonists act directly at the pituitary level to block GnRH receptor signalling, leading to a rapid reduction of LH and FSH secretion. Because ovarian androgen synthesis is acutely LH-dependent, this results in an immediate reduction in ovarian androstenedione production. In contrast, adrenal steroidogenesis and peripheral conversion are not directly regulated by the pituitary–gonadal axis, explaining why circulating testosterone levels exhibit a less pronounced response to GnRHR antagonist administration.

Together, these findings show that carefully titrated GnRHR antagonism with Ganirelix appears to achieve an optimal balance, sufficiently suppressing LH to alleviate hyperandrogenism. However, it should be acknowledged that the absence of a placebo or healthy comparator arm in the present study precludes definitive quantification of the magnitude of endocrine changes and does not allow formal exclusion of nonspecific gonadotrophin suppression; placebo-controlled trials will therefore be essential to establish the full specificity and extent of the neuroendocrine effects observed here.

While participants with BMI >30 kg/m^2^ were deliberately excluded from this study to isolate the direct neuroendocrine effects of Ganirelix from the confounding influence of obesity-associated insulin resistance, which exacerbates hyperandrogenism through reduction of SHBG levels,^4^ this necessarily limits the generalisability of our findings to the broader PMOS population. Nevertheless, women with PMOS with obesity also exhibit neuroendocrine disturbances including elevated LH pulsatility,^10^^,^^12^ suggesting that this subgroup may similarly benefit from GnRHR antagonist therapy, a hypothesis that future trials enrolling more phenotypically diverse populations will need to address. Beyond the exclusion of participants with BMI >30 kg/m^2^, no comprehensive metabolic profiling was performed in this study; parameters such as fasting insulin, HOMA-IR, and lipid profile were not collected, and it is therefore possible that a subset of enrolled participants harboured underlying insulin resistance or other metabolic disturbances. However, given the acute and direct mechanism of GnRHR competitive blockade, which operates at the pituitary level independently of metabolic pathways, such disturbances are unlikely to have substantially influenced the neuroendocrine response to Ganirelix over the 4-h study window following the injection; nonetheless, future trials should include systematic metabolic characterisation of participants to formally address this question.

The above considerations are particularly relevant when interpreting the limited clinical literature currently available on the use of GnRHR antagonists in PMOS.

With the exception of IVF protocols, clinical studies on the use of GnRHR antagonists in PMOS remain scarce. A recent trial tested oral Elagolix at varying doses versus placebo for 6 months in women with PMOS, evaluating cycle patterns as well as LH, FSH, oestradiol, and progesterone levels.^69^ Although Elagolix lowered LH and testosterone levels, it did not restore ovulation, menstrual cycles remained irregular and progesterone concentrations stayed low. In addition, only 34 of the 114 participants completed the trial. Importantly, Elagolix differs fundamentally from Ganirelix and Cetrorelix because it is a non-peptide, orally active GnRHR antagonist with distinct pharmacokinetic and pharmacodynamic characteristics.^39^ In contrast to the injectable peptide antagonists used in our study, Elagolix undergoes rapid oral absorption and systemic clearance, producing a different pattern of GnRH receptor occupancy and pituitary suppression. These pharmacological differences may therefore contribute to the distinct reproductive outcomes reported with Elagolix compared with the restoration of ovulation and cyclicity observed in our preclinical low-dose Ganirelix paradigm.

Moreover, the doses administered (50–300 mg/day) were likely excessive, given that 150 mg/day, used for endometriosis-associated pain and heavy menstrual bleeding, already exerts a suppressive effect on gonadotrophins.^70^ This interpretation aligns with the observed reduction in oestradiol across all Elagolix-treated groups. Notably, the study did not evaluate whether these doses normalised LH pulsatility in women with PMOS.

In recent years, additional neuroendocrine strategies are being explored as potential new therapeutic avenues for PMOS.^64^ Kisspeptin neurons in the arcuate nucleus (infundibular nucleus in humans), which co-express neurokinin B (NKB) and dynorphin and are collectively termed KNDy neurons, play a central role in regulating pulsatile GnRH release^71^ through auto/paracrine signalling via the NKB receptor NK3R. Consequently, blockade of NKB signalling has been explored as an indirect strategy to attenuate LH hypersecretion and hyperandrogenism in women with PMOS.^71^ The NK3R antagonist MLE4901 reduced LH pulsatility and testosterone levels in women with PMOS but was discontinued due to hepatotoxicity.^72^^,^^73^ More recently, Fezolinetant similarly reduced LH, the LH/FSH ratio, and androgen levels in women with PMOS while maintaining oestradiol and progesterone concentrations.^74^ However, no clear restoration of ovulatory cyclicity was observed during the study period,^74^ suggesting that the degree or pattern of neuroendocrine suppression achieved may not have been sufficient to fully restore reproductive function. One possible explanation is that, although KNDy neurons in the arcuate nucleus are critical for generating GnRH pulsatility, kisspeptin neurons in the preoptic area are thought to mediate the midcycle LH surge.^75^^,^^76^ Consequently, while NK3R antagonists may initially provide therapeutic benefit by lowering LH secretion, they could also suppress the preovulatory LH surge required for ovulation.

At the same time, growing recognition of hyperandrogenism as a key factor disrupting oestrogen and progesterone negative feedback on GnRH neurons, through downregulation of ERα and progesterone receptor expression, has renewed interest in anti-androgen therapies such as flutamide. In preclinical studies, using both lean and metabolically impaired PMOS-like mouse models, flutamide treatment restored oestrous cyclicity and improved reproductive function.^24^^,^^77^^,^^78^ However, despite promising preclinical efficacy, the clinical use of flutamide in PMOS is limited by severe hepatotoxicity.^9^

Beyond strategies targeting androgen action or neuroendocrine dysfunction, recent preclinical research has also pointed to normalise the aberrant AMH-AMHR2 signalling as a potential therapeutic target in PMOS pathophysiology.^37^^,^^79^

Together, these emerging approaches highlight the growing interest in therapies aimed at correcting the upstream neuroendocrine and endocrine mechanisms driving PMOS rather than solely treating downstream symptoms.

Within this broader therapeutic context, the present clinical study provides initial evidence that low-dose GnRHR antagonism can selectively modulate the neuroendocrine abnormalities characteristic of women with PMOS.

However, because this was a small exploratory phase I study designed primarily to assess short-term endocrine responses, the present data do not allow conclusions regarding restoration of ovulation, normalisation of menstrual cyclicity, or improvement in fertility outcomes. In addition, the multiple secondary endocrine endpoints analysed in this study were evaluated without correction for multiple comparisons. Consequently, these findings should be considered hypothesis-generating and will require confirmation in larger, adequately powered, placebo-controlled studies specifically designed to evaluate long-term reproductive and clinical outcomes.

Overall, our results provide a rationale for future phase II clinical trials investigating the safety, long-term efficacy, and reproductive effects of partial and carefully titrated gonadotrophin-releasing hormone receptor blockade in PMOS.

## Contributors

Conceptualisation, P.G.; writing and preparation of the figures, L.C. and P.G.; L.C., S.C-J. and P.G. analysed and verified the underlying data; methodology and formal analysis, L.C., H.M., F.G., S.B., P.P.; F.G. performed the measurements of mouse steroid levels in serum by GC-MS/MS; H.M., G.R., S.C-J., performed the clinical study; P.P. performed the measurements of sex steroids, gonadotrophins and AMH in serum of women; review/editing, P.G., L.C, H.M., F.G., S.B., P.P., G.R., S.C-J.; and funding acquisition, P.G. All authors read and approved the final version of the manuscript.

## Data sharing statement

De-identified participant data underlying the results reported in this article will be made available to qualified researchers upon reasonable request to the corresponding author. Requests should be submitted by email and include a brief description of the research objectives and planned analyses. Data will be shared solely for academic, non-commercial purposes following review of the proposal and, where applicable, completion of a data-sharing agreement. No identifying information will be disclosed, and all shared datasets will comply with applicable ethical, privacy, and data-protection regulations. The study protocol, pre-clinical data, raw data, and data analyses generated during the study will also be available upon request. All data will be accessible from the date of publication of the manuscript by contacting the corresponding author.

## Declaration of interests

P.G. discloses that he is an inventor of a patent application by the INSERM (Institut National de la Santé et de la Recherche Médicale) covering the use of GnRH antagonists for the treatment of women affected with PMOS (N° WO2018177746; US patent N°: US 12,208,130 B2). All other authors do not have competing interests.

## Appendix A Supplementary data

Supplementary Figure

Protocol
