# Supplementary material for: GnRH-receptor antagonism as a targeted approach to reproductive dysfunction in polycystic ovary syndrome
Source: eBioMedicine. 2026 Jul 17;130:106398. doi: 10.1016/j.ebiom.2026.106398 (PMC13400290; doi:10.1016/j.ebiom.2026.106398)
Supplement: Supplementary Figure [file mmc1.pdf]

SUPPLEMENTARY MATERIAL

GnRH-receptor Antagonism as a Targeted Approach to Reproductive Dysfunction in Polycystic Ovary Syndrome

Ludovica Cotellessa, Hélène Maitre, Frank Giton, Silvia Bongiovanni, Pascal Pigny, Geoffroy Robin, Sophie Catteau-Jonard and Paolo Giacobini

Supplementary material, PDF file containing Supplementary Figure 1.

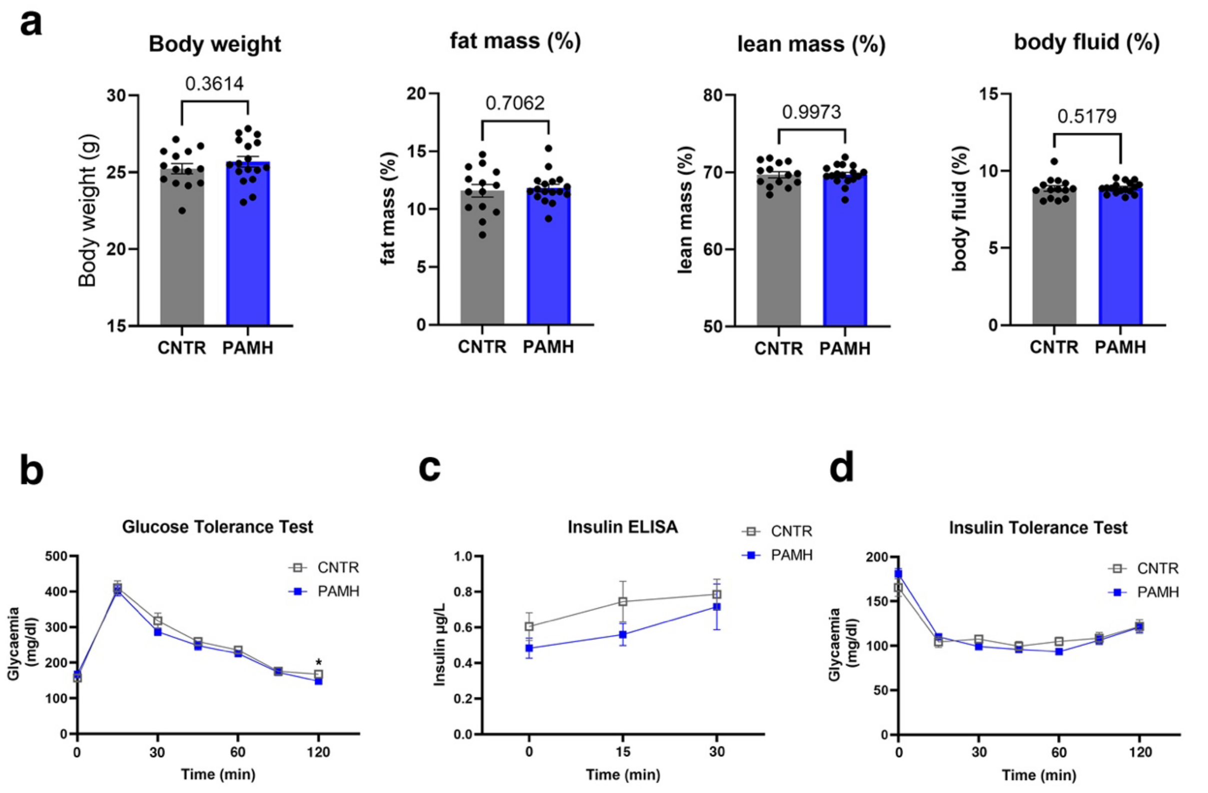

Supplementary Figure 1. Metabolic characterization of 4-month-old PAMH female mice. (**a**) Body weight and body composition parameters, including fat mass, lean mass, and body fluid percentage, in control (CNTR; *n* = 14) and PAMH (*n* = 17) female mice at 4 months of age. (**b**) Blood glucose levels measured during a glucose tolerance test (GTT) in CNTR (*n* = 7) and PAMH (*n* = 7) female mice at 4 months of age. (**c**) Serum insulin concentrations measured during the first 30 min of the GTT in CNTR (*n* = 7) and PAMH (*n* = 7) female mice at 4 months of age. (**d**) Blood glucose levels measured during an insulin tolerance test (ITT) in CNTR (*n* = 7) and PAMH (*n* = 7) female mice at 4 months of age.

Data are presented as mean ± SEM. Statistical analyses were performed using an unpaired Student's t-test for panel (**a**) and two-way ANOVA followed by Tukey's multiple-comparisons testing for panels (**b-d**); $*p < 0.05$.
