# Supplementary material for: GnRH-receptor antagonism as a targeted approach to reproductive dysfunction in polycystic ovary syndrome
Source: eBioMedicine. 2026 Jul 17;130:106398. doi: 10.1016/j.ebiom.2026.106398 (PMC13400290; doi:10.1016/j.ebiom.2026.106398)
Supplement: Protocol [file mmc2.docx]

**Sub-therapeutic GnRH- Antagonist Treatment to Rectify LH Pulsatility in Lean Women With PCOS**

***(SOPKGANI)***

| **Code Number assigned** | **2022_0016** |
| --- | --- |
| **N° EudraCT** | **2023-000176-35** |
| **Research involving humans** | **Research category 1 : Interventional study** |
| **Coordinator** | **Pr Sophie Catteau-Jonard**, PU-PH, RPPS 10002305695  Hospital Jeanne de Flandre- Medical Gynaecology Service  Address : av. Eugène Avinée CHU Lille, 59000 Lille  Tel: 03 20 44 66 18 Fax : 03 20 44 64 07  e-mail : sophie.catteau@chru-lille.fr |
| **Scientific collaborator** | **Dr Paolo Giacobini**  INSERM U1172 Centre JP Aubert CHU Lille  Mail : paolo.giacobini@inserm.fr |
| **Promoter** | **University Hospital Center of Lille**  Department of Research and Innovation (DRI)  6 rue Paul Laguesse  59037 LILLE Cedex  tél : 03 20 44 59 69 |
| **Methodologist** | **Ariane Leroyer** |
| **Financial support** | **Horizon-ERC-POC grant (ERC-2022-POC2 to P.G., grant agreement n° 101111874/GRASP)** |
| **Number Clinical Trial (NCT)** | **NCT05751252** |
| **N° Version en cours/ Date :** | **Version n°1.0 du 30/11/2022** |

**LISt of the recruiting centers**

| **n°** | **site** | **PRINCIPAL INVESTIGATOR** |
| --- | --- | --- |
| **1** | **Jeanne de Flandre** | **Pr S Catteau-Jonard** |
|  |  |  |

**ProTOCOL SIGNATURE PAGE**

**Sponsor code :** 2022_0016

**Full/short title :** Sub-therapeutic GnRH- Antagonist Treatment to Rectify LH Pulsatility in Lean Women With PCOS

**Version number and date :** n°1 du 30/11/2022

The coordinator and the sponsor undertake to carry out this study in accordance with the protocol, the rules and recommendations of international Good Clinical Practice (GCP), and the legislative and regulatory provisions applicable to research.

| **SPONSOR**  **LEGAL REPRESENTATIVE** | **CHU de Lille**  **M Frédéric Boiron** | **DATE**  **_ _ / _ _ / _ _ _ _** | **SIGNATURE** |
| --- | --- | --- | --- |
| **COORDINATOR/RESPONSIBLE** | **Pr Sophie CATTEAU-JONARD** | **DATE**  **_ _ / _ _ / _ _ _ _** | **SIGNATURE** |

**SIGNATURE OF THE COORDINATOR/PRINCIPAL INVESTIGATOR**

I have read all pages of this protocol, for which the Lille University Hospital (CHU de Lille) is the sponsor, and I confirm that it contains all the information necessary for the conduct of the trial.

I undertake to carry out the trial in compliance with the protocol and the terms and conditions defined therein, as well as any amendments that may be transmitted to me by the sponsor. I commit to conducting this protocol in accordance with Good Clinical Practice (GCP), the French Public Health Act of August 9, 2004, and the implementing decree of November 16, 2016, in particular by providing information to the patients and obtaining their written consent before any protocol-related screening procedure.

I also undertake to ensure that the investigators and other qualified members of my team have access to copies of this protocol and to all documents related to the conduct of the trial, so that they may perform their work in compliance with the provisions contained in these documents.

| **PRINCIPAL INVESTIGATOR**  **HOSPITAL** | **Pr Sophie CATTEAU-JONARD**  **Lille University Hospital, Jeanne de Flandre Hospital, Department of Medical Gynecology** | **DATE**  **_ _ / _ _ / _ _ _ _** | **SIGNATURE** |
| --- | --- | --- | --- |

## ****List of Abbreviations****

| **Abbreviation** | **Definition** |
| --- | --- |
| **ANSM** | National Agency for the Safety of Medicines and Health Products (Agence Nationale de Sécurité du Médicament) |
| **CA** | Competent Authority |
| **CRA** | Clinical Research Associate |
| **IB** | Investigator’s Brochure |
| **GMP** | Good Manufacturing Practices |
| **CHU** | University Hospital Center |
| **CNIL** | National Commission on Informatics and Liberty |
| **CPP** | Ethics Committee / Committee for the Protection of Persons |
| **CRF / eCRF** | Case Report Form / electronic Case Report Form |
| **INN** | International Nonproprietary Name |
| **AE** | Adverse Event |
| **SAE** | Serious Adverse Event |
| **PI** | Principal Investigator |
| **SmPC** | Summary of Product Characteristics |
| **VRB** | Volunteers for Biomedical Research |

**Full study synopsis**

| **SPONSOR** | University Hospital Center (CHU) of Lille |
| --- | --- |
| **LONG TITLE** | Sub-therapeutic GnRH-Antagonist Treatment to Rectify LH Pulsatility in Lean Women With PCOS |
| **SHORT TITLE** | GANIRELIX AND PCOS |
| **ACRONYM** | SOPKGANI |
| **STUDY CORDINATOR** | Prof. S. Catteau-Jonard, MD, PhD, Department of Medical Gynecology, CHU de Lille |
| **MEDICAL CONDITION** | Polycystic Ovary Syndrome (PCOS) in women with AMH > 28 pmol/L, LH > 8 IU/mL, and serum testosterone > 0.39 ng/mL |
| **KEYWORDS** | PCOS; GnRH antagonist; LH; testosterone; androgens; FSH |
| **NUMBER OF CENTERS** | 1 (monocentric) |
| **SCIENTIFIC RATIONALE** | We will conduct a clinical study using low doses of a GnRH antagonist (Ganirelix) to reduce the abnormally high LH pulsatility in women with PCOS. By decreasing LH levels, we anticipate a reduction in androgen concentrations. The goal is to demonstrate that low-dose GnRH antagonists can reduce LH pulsatility by 20–30%, achieving values comparable to healthy women, and lower androgen levels by at least 20–30% without suppressing the hypothalamic-pituitary-gonadal axis or reproductive functions. |
| **PLAN EXPERIMENTAL** | **Pilot, phase 1, monocentric study with two parallel groups**, evaluating the efficacy of two doses of Ganirelix in reducing the frequency, mean and basal LH secretion in women with PCOS, in order to reach values comparable to those observed in healthy subjects (approximately 20–30% reduction).  The **European Medicines Agency (EMA)** medical report on Ganirelix safety data indicates no specific risk to humans based on conventional studies of safety pharmacology, repeated-dose toxicity, genotoxicity, carcinogenic potential, and reproductive toxicity. **Ganirelix is generally well tolerated**, even at higher concentrations (0.25 mg dose).  Participants included in the study will undergo **LH measurement every 10 minutes for 8 hours**, according to two phases:   - A **pre-treatment phase** of 4 hours; - A **post-treatment phase** of 4 hours, during which patients will receive one of the two Ganirelix doses.   **Androgens, FSH, AMH, and estradiol** will be measured at the start of the procedure (H0) and at the end (H8). |
| **STUDY ARMS AND TREATMENTS** | **Two experimental groups will be studied:**   - **Experimental Group 1:** Participants will be monitored for 4 hours without treatment, then will receive a dose of **0.0625 mg of Ganirelix**, followed by 4 additional hours of monitoring after injection. - **Experimental Group 2:** Participants will be monitored for 4 hours without treatment, then will receive a dose of **0.0250 mg of Ganirelix**, followed by 4 additional hours of monitoring after injection. |
| **STUDY PHASE** | Phase 1 |
| **OBJECTIVES AND EVALUATION CRITERIA** | ****Primary Objective**** To evaluate the effect of Ganirelix on the reduction of LH secretion frequency using two different doses. ****Primary Endpoint**** Aiming for a **20–30% reduction in LH pulse frequency** during the 4 hours following injection, to reach values comparable to those observed in healthy women. To be considered an LH pulse or peak, the value had to show an increase of more than 20% compared with one of the two preceding points, followed by a decrease of more than 10% in one of the two subsequent points. ****Secondary Objective No. 1**** To assess the variation in LH secretion (mean and basal secretion) before and after Ganirelix injection.  **Secondary Endpoint No. 1:** The variation in LH secretion before and after Ganirelix injection will be calculated as the mean LH (the average of all LH values over the study period) and basal LH, corresponding to the mean of the five lowest LH values over the study period. The total LH corresponds to the sum of all LH values at T0-T240 and T240-T480. ****Secondary Objective No. 2**** To evaluate the effect of two different doses of Ganirelix on androgen production.  **Secondary Endpoint No. 2:** Variation in androgen production 4 hours after injection, defined as the difference between total testosterone and androstenedione levels measured at H8 and H0 (4 hours before injection). ****Secondary Objective No. 3**** To evaluate the concentrations of FSH, estradiol, and AMH under low-dose Ganirelix at H0 and H8, before and after treatment.  **Secondary Endpoint No. 3:** Difference between the mean concentrations of FSH, estradiol, and AMH measured at H0 and H8. |
| **STUDY POPULATION** | ****Inclusion Criteria****  - Women aged **18 to 35 years** - Diagnosis of **PCOS** with **AMH > 28 pmol/L**, **LH > 8 IU/mL**, and **serum testosterone > 0.39 ng/mL** - **No hormonal treatment or contraception** for at least **2 months** prior to inclusion - **No treatment with Metformin** or any other medication affecting metabolism - **Affiliated with the French Social Security system** - **Body Mass Index (BMI)** below 30 **kg/m²**  ****Exclusion Criteria****  - **Pregnant women** - **Inability to understand the information leaflet** - **Known hypersensitivity** to Ganirelix, to any structural analog of gonadotropin-releasing hormone (GnRH), to exogenous peptide hormones, or to any of the excipients |
| **SAMPLE SIZE** | 20 women |
| **STATISTICAL ANALYSIS PLAN** | **General Principles** All statistical analyses will be performed independently by the Department of Biostatistics, University of Lille, under the supervision of Mr. Julien Labreuche. Analyses will be conducted using 10.0.2 GraphPad Prism software (GraphPad; San Diego, CA, USA).  All analyses will be performed on the per-protocol population, unless otherwise specified. The level of statistical significance will be set at α = 0.05 (two-sided). No adjustment for multiple testing will be applied, and all secondary analyses will be considered exploratory. Results will be presented as effect size estimates with corresponding 95% confidence intervals (95% CI).  **Description of Data** Baseline demographic and clinical characteristics will be described by treatment group. Quantitative variables will be summarized as mean (standard deviation or standard error). The normality of data distributions will be evaluated using the Shapiro–Wilk test.  For normally-distributed data, data will be compared using unpaired two-sided Student’s *t*-test. For non-normally distributed values, Mann-Whitney U test will be used.  **Primary Endpoint Analysis** The primary endpoint is the reduction in LH pulse frequency during the 4-hour period following Ganirelix administration. Analysis will include: - Determination of LH pulse frequency using the method described above (1.1.2).  **Secondary Endpoint Analyses** - LH variation (mean, basal and total LH) before and after Ganirelix injection will be assessed. - Androgen production variation will be evaluated as the difference in total testosterone and androstenedione levels between H8 and H0 (4 hours post- vs. pre-injection). - FSH, estradiol, and AMH concentrations will be compared between H0 and H8 to assess hormonal changes after treatment.  **Handling of Missing Data** Given the short study duration and intensive monitoring, missing data are expected to be minimal. Any missing measurements will be described, and no imputation is planned. Analyses will be based on available data only.  **Statistical Software** All statistical analyses will be performed using: 10.0.2 GraphPad Prism software (GraphPad; San Diego, CA, USA)  **Justification for the Absence of a Power Calculation** As this is an **exploratory study** no formal sample size calculation or statistical power analysis has been performed. The main objective of this research is to generate preliminary physiological and pharmacodynamic data on the effect of low-dose Ganirelix on LH pulsatility in women with PCOS, rather than to formally test a hypothesis. Given the mechanistic and proof-of-concept nature of the study, the selected sample size of 20 participants is deemed appropriate to: - Evaluate feasibility and study logistics; - Provide initial estimates of variability in LH pulse frequency and amplitude under low-dose GnRH antagonist treatment; - Support the design of a future adequately powered confirmatory trial. This approach is consistent with international regulatory guidance for early-phase exploratory studies (ICH E9, EMA guidelines on exploratory trials), which emphasize estimation over formal hypothesis testing in pilot settings. |
| **STUDY TIMELINE** | Recruitment: 12 months Participant duration: 1 week Total study duration: 12 months + 1 week |
| **STUDY-SPECIFIC INVESTIGATION PROCEDURE AND DIFFERENCES COMPARED TO STANDARD CARE** | Standard treatment of endometriosis and stimulation protocols for IVF. Its use at a low dose is not common, nor is its use in women with PCOS (outside the context of endometriosis or an IVF protocol) |
| **ASSESSMENT OF THE BENEFITS AND RISKS RELATED TO THE RESEARCH** | **No individual benefit is guaranteed.**  **Risks related to the treatments:** A possible allergy to Ganirelix may occur, presenting as redness, warmth of the skin, itching, hives, nasal discharge, rapid or irregular pulse, swelling of the tongue and throat, sneezing, wheezing or significant breathing difficulties, or dizziness. Particular caution should be exercised in women showing signs or symptoms of an active allergic condition or with a known history of allergic predisposition. Treatment with Ganirelix is not recommended for women suffering from severe allergic conditions.  **Other risks described in the Summary of Product Characteristics (SmPC):**   - Skin irritation at the injection site: redness, itching, swelling; - Nausea; - Headaches.   **Risks related to blood sampling:** Blood collection may cause very slight discomfort, as samples are taken frequently, and minor bruising may appear at the site for a few days after the blood draw. |
| **SIMULTANEOUS PARTICIPATION IN ANOTHER STUDY** | **NO.** Participants enrolled in this study shall not, for the duration of the study, take part concurrently in any other interventional clinical trial involving investigational or marketed medicinal products. |
| **EXCLUSION PERIOD FOLLOWING THE STUDY** | **NO.** |
| **JUSTIFICATION FOR WHETHER OR NOT A MONITORING COMMITTEE IS ESTABLISHED** | In view of the existing data on Ganirelix and the low doses administered to patients enrolled in this study, the establishment of a Safety Monitoring Committee is deemed unnecessary. |
| **FUNDINGS SOURCES** | Horizon-ERC-POC grant (ERC-2022-POC2 to P.G., grant agreement n° 101111874/GRASP) |
| **HEALTH TECHNOLOGY MATURITY LEVEL (TRL)** | TRL 6c |

**SYNOPSIS FOR « ClinicalTrials.gov**. **» REGISTRATION**

x **Study Type** **Interventional:** *studies in human beings in which individuals are assigned by an investigator based on a protocol to receive specific interventions. Subjects may receive diagnostic, therapeutic or other types of interventions. The assignment of the intervention may or may not be random. The individuals are then followed and biomedical and/or health outcomes are assessed.*

| **Official Title** | Short-term GnRH- antagonist treatment to lower LH pulsatility in women with PCOS aiming to improve hormonal functions |
| --- | --- |
| **Brief Title** | Sub-therapeutic GnRH- antagonist treatment to rectify LH pulsatility in lean women with PCOS. |
| **Acronym** | SOPKGANI |
| **Collaborators** | ERC GRASP (PoC) |
| **Brief summary** | We will conduct a clinical study using a sub-therapeutic dose of a GnRH antagonist to reduce overactive LH pulsatility in women with PCOS. With the intervention and lowered LH action we anticipate to decrease androgen levels in women with PCOS. We aim to show for the first time that low-dose GnRH-antagonists can lower LH pulsatility by 20–30% and decrease androgen levels without blunting the hypothalamic-pituitary-gonadal axis and thereby the reproductive functions. |
| **Conditions** | Polycystic Ovary Syndrome |
| **Keys Words** | PCOS ; GnRH antagonist ; LH ; androgen ; testosterone |
| **Primary purpose** | Treatment  Prevention  Diagnostic  Supportive care  Screening  Health services Research  Basic science  Other |
| **Intervention model** | Single group  Parallel  Crossover  Factorial |
| **Study classification** | Safety  Safety/Efficacy  Efficacy  Bio-equivalence  Bio-availability  Pharmacokinetics  Pharmacokinetics/dynamics  Pharmacodynamics |
| **Interventions (repeating as many times as arms)** | |
| **Name of arm 1** | Pilot phase-0, parallel, clinical study.  Test the efficacy of Ganirelix administered subcutaneously at single-dose regimen of 0.0625 mg in 10 women with PCOS, in rectifying LH pulse frequency and LH amplitude. |
| **Intervention (at least one intervention)** | Drug (including placebo)  Radiation  Other  Device (including sham) Behavioral  Biological / vaccine  Genetic  Procedure / surgery  Dietary supplement |
| **Intervention name** | Ganirelix (Orgalutran) |
| **Intervention description** | An intra-venous cannula will be inserted and blood will be sampled at 10-min intervals for a 4-h baseline period commencing at 0800 h. Ganirelix will be then administered subcutaneously at single-dose regimen at 0.0625 mg (n = 10 women with PCOS). After Ganirelix administration sampling will continue at 10-min intervals for 4 h. |
| **Name of arm 2** | Pilot phase-0, parallel, clinical study.  Test the efficacy of Ganirelix administered subcutaneously at single-dose regimen of 0.025 mg in 10 women with PCOS, in rectifying LH pulse frequency and LH amplitude. |
| **Intervention (at least one intervention)** | Drug (including placebo)  Radiation  Other  Device (including sham) Behavioral  Biological / vaccine  Genetic  Procedure / surgery  Dietary supplement |
| **Intervention name** | Ganirelix (Orgalutran) |
| **Intervention description** | An intra-venous cannula will be inserted and blood will be sampled at 10-min intervals for a 4-h baseline period commencing at 0800 h. Ganirelix will be then administered subcutaneously at single-dose regimen at 0.025 mg (n = 10 women with PCOS). After Ganirelix administration sampling will continue at 10-min intervals for 4 h. |
|  | **Primary Outcome measure** |
| **Title** | Serum LH level every 10 minutes for 8 hours |
| **Time point at which primary outcome measure is assessed** | The degree of gonadotropin suppression will be determined by calculating the percent inhibition from the pre-antagonist period [(mean PRE - nadir)/mean PRE] x 100, where nadir hormone levels will be calculated using a moving average. |
|  | **Secondary outcome measures, repeating as many times as 2nd outcomes** *Noter les 5 principaux critères d’évaluation* |
| **Title** | Measurement of serum testosterone and androstenedione, FSH, estradiol and AMH levels |
| **Time point(s) at which Secondary outcome measure is assessed** | Serum testosterone and androstenedione, FSH, AMH and estradiol levels at the beginning and at the end of the 8 hours |
| **Inclusion criteria** | -women aged 18 to 35  -women with PCOS with AMH> 28 pmol / L, LH> 8 IU / mL and testosteronemia > 0.39 ng/mL  - no hormonal treatment or contraception for 2 months  - women covered by the Social Security system  Non obese women |
| **Exclusion criteria** | -hormonal treatment or hormonal contraception  - Metformin treatment  -pregnant woman  -inability to understand the newsletter |

# SCIENTIFIC RATIONALE

### ****Polycystic Ovary Syndrome (PCOS)****

Polycystic Ovary Syndrome (PCOS) is the most common reproductive and metabolic disorder, affecting up to 18% of women worldwide, and is responsible for about 70% of cases of anovulatory infertility¹^,^². This endocrine disorder is present throughout a woman’s life, has a noticeable impact on quality of life, and represents a significant clinical, psychological, and economic “burden.” It is frequently associated with considerable medium- and long-term morbidities: pregnancy-related complications, placental dysfunction, increased obstetric morbidity (gestational diabetes, preeclampsia, preterm birth), obesity, type 2 diabetes, hepatic steatosis, and an increased risk of neuropsychiatric disorders ³.

In the United States alone, health care expenditures related to PCOS among women of reproductive age were estimated to exceed $5 billion per year in 2019 ⁴. Comparable data are not available for Europe, but it is likely that the health care costs associated with PCOS exceed €1 billion annually.

The diagnostic criteria for PCOS have been revised several times — by the Rotterdam consensus, the Androgen Excess and PCOS (AE-PCOS) Society, and the National Institutes of Health (NIH). There is no universally accepted version, although an international evidence-based guideline for the assessment and management of PCOS⁵ endorses the use of the Rotterdam diagnostic criteria. According to these criteria, a woman must meet two of the following three criteria for a diagnosis of PCOS: oligomenorrhea or amenorrhea; biochemical or clinical evidence of hyperandrogenism (specifically hirsutism and acne); and typical ultrasound features of polycystic ovaries. The Rotterdam criteria yield four PCOS phenotypes:

- **Type A (classic PCOS):** hyperandrogenism (HA) + irregular/absent menstrual cycles (ovulatory dysfunction, OD) + polycystic ovarian morphology (PCOM);
- **Type B:** HA + OD;
- **Type C:** HA + PCOM;
- **Type D:** OD + PCOM.

Elevated androgens represent a major feature of PCOS, as the majority (nearly 80%) of women with PCOS have hyperandrogenism (Rotterdam definition) ⁶. Moreover, most women (75%) diagnosed with PCOS show accelerated LH pulsatility, independent of obesity status ⁷^,^⁸, suggesting a rapid frequency of GnRH pulsatility ⁹^,^¹⁰.

Although the defining features of PCOS (i.e., androgen excess, oligo-/anovulation, and polycystic ovarian morphology) are most directly related to ovarian function, the central reproductive neuroendocrine system — the gonadotropin-releasing hormone (GnRH) — ultimately determines ovarian function through its regulation of gonadotropin release. Altered GnRH secretion plays a significant role in the pathophysiology of PCOS ¹¹.

Previous work by the Giacobini group has shown that anti-Müllerian hormone (AMH) induces LH secretion by stimulating activation and secretion of neuropeptides in GnRH neurons ¹², which express AMH receptors in both rodents and humans ¹²-¹⁴. AMH is secreted by granulosa cells of ovarian follicles ¹⁵ and regulates early follicular development by influencing the transition from resting primordial follicles to growing follicles ¹⁶.

Earlier studies have shown that plasma AMH levels in patients with PCOS are 2–3 times higher than those in women with normal ovaries ¹⁷^,^¹⁸, and that the severity of the PCOS phenotype correlates with AMH production — higher in anovulatory PCOS patients than in ovulatory ones ¹⁹^,^²⁰. Furthermore, the Giacobini and Piltonen groups have shown in two collaborative studies that elevated AMH levels persist throughout pregnancy in women with PCOS compared with gestational-age– and weight-matched non-PCOS controls ¹⁴^,^²¹.

Animal models have contributed to a better understanding of the etiopathogenesis of PCOS and its underlying pathophysiological mechanisms, confirming the central role of hyperandrogenism in the development of this condition. Indeed, PCOS models generated by inducing hyperandrogenism consistently produce animals exhibiting a wide range of reproductive, endocrine, and metabolic features of PCOS ¹. Prenatal exposure to testosterone or early postnatal exposure to dihydrotestosterone (DHT) has been reported to generate a PCOS-like phenotype in rodents (the PNA model), as well as in sheep and non-human primates ¹.

Recent studies from the Giacobini group have implicated AMH signaling in the brain as a potential player in the development and pathophysiology of PCOS ¹⁴^,^²². Pregnant mice injected with AMH showed increased testosterone production, reduced placental conversion of testosterone to estradiol, and offspring (the PAMH model) predisposed to PCOS-like features after puberty ¹⁴. These studies suggest that prenatal AMH treatment promotes remodeling of placental steroidogenesis, leading to inhibition of aromatase expression and thereby increasing in utero testosterone bioavailability. These findings are consistent with clinical investigations showing reduced placental aromatase activity in women with PCOS ²³.

AMH-induced androgenization may also rely on central AMH actions that enhance GnRH release, since PAMH mothers also display elevated LH and testosterone levels at term ¹⁴, similar to findings in humans ²¹.

We have observed that PCOS-like females exhibit persistent hyperactivity of GnRH neurons and that treatment with a GnRH antagonist in adult female offspring restores their neuroendocrine phenotype to normal. These findings highlight the critical role of excessive prenatal AMH exposure and hyperstimulation of GnRH receptors in the neuroendocrine dysfunctions of PCOS, while offering a new potential therapeutic avenue for treating this condition in adulthood.

It is important to note that in women with PCOS, long-acting GnRH agonists — which suppress gonadotropin secretion — significantly reduce circulating androgen concentrations. Likewise, suppression of gonadotropins partly explains the effectiveness of combined oral contraceptives in treating PCOS-related hyperandrogenism ²⁴.

Based on the above preclinical and clinical evidence, and the established safety of GnRH antagonists (FDA-approved), we are confident that a clinical trial using a low-dose GnRH antagonist to reduce hyperactive LH pulsatility in women with PCOS should yield significant beneficial effects associated with normalization of abnormal androgen and LH levels.

# STUDY OBJECTIVES

### ****3.1 Primary Objective****

To assess the effect of Ganirelix on the reduction of luteinizing hormone (LH) pulse frequency, comparing two different dose levels.

### ****3.2 Secondary Objectives****

**Secondary Objective 1:**
To evaluate the effect of two dose levels of Ganirelix on LH secretion (mean, basal and total).

**Secondary Objective 2:**
To assess the effect of two dose levels of Ganirelix  on LH variation (mean, basal and total LH) before and after Ganirelix injection will be assessed.

To assess the effect of two dose levels of Ganirelix on androgen production.

**Secondary Objective 3:**
To determine the effect of two dose levels of Ganirelix on variations in follicle-stimulating hormone (FSH), estradiol, and anti-Müllerian hormone (AMH) levels.

# EVALUATION CRITERIA

### ****4.1 Endpoint for the Primary Objective****

The expected decrease in LH pulse frequency before and after Ganirelix* administration is estimated to be between 20% and 30%.
To be considered an LH pulse or peak, the value had to show an increase of more than 20% compared with one of the two preceding points, followed by a decrease of more than 10% in one of the two subsequent points.

### ****4.2 Endpoints for the Secondary Objectives****

**Secondary Endpoint 1:**
The variation in LH secretion before and after Ganirelix injection will be calculated as the mean LH (the average of all LH values over the study period) and basal LH, corresponding to the mean of the five lowest LH values over the study period. The total LH corresponds to the sum of all LH values at T0-T240 and T240-T480.

**Secondary Endpoint 2:**
Variation in androgen production at 4 hours post-injection, defined as the difference between the total testosterone and androstenedione levels measured at H8 (4 hours after injection) and H0 (4 hours before injection).

**Secondary Endpoint 3:**
Variation in FSH, estradiol, and AMH levels at 4 hours post-injection, defined as the difference between concentrations measured at H8 (4 hours after injection) and H0 (4 hours before injection).

# METHODOLOGY

### ****5.1 Study Design****

This is a **single-center, phase I pilot study** with **two parallel groups**.

The study will be proposed during endocrinological gynecology consultations to all patients meeting the inclusion criteria. After obtaining written informed consent, participants will be assigned to one of the two dose groups.

During the 4 hours preceding Ganirelix injection*, baseline LH secretion and pulsatility, as well as baseline levels of androgens, FSH, estradiol, and AMH, will be measured.

Subsequently, patients will receive a **low-dose injection of Ganirelix***. LH secretion and pulsatility will be monitored for **4 hours following the injection**, along with measurements of androgens, FSH, estradiol, and AMH at **H8**.

The expected outcome is to identify the Ganirelix* dose that reduces the abnormal frequency of LH pulses in women with PCOS to values comparable to normal conditions (approximately a 30% reduction).

As **secondary outcomes**, reproductive markers (serum levels of testosterone, androstenedione, LH, FSH, estradiol, and AMH) as well as glucose, insulin, and liver function parameters will be assessed before and after the 4-hour GnRH antagonist treatment period.

### ****5.2 Measures Taken to Minimize Bias****

Each participant will serve as her own control in this study, thereby minimizing potential bias.

The phenotypic diversity among women with PCOS — particularly regarding lean versus obese status — has a significant impact on mean plasma LH levels. To reduce variability, this pilot study will focus exclusively on **lean women with a highly uniform PCOS phenotype**, defined as follows:

- Age between **18 and 35 years**,
- **BMI below 30 kg/m²**,
- **Hyperandrogenism** (total testosterone > 0.39 ng/mL),
- **Elevated plasma LH** (> 8 IU/mL),
- **Elevated plasma AMH** (> 28 pmol/L).

#### ****5.2.1 Randomization****

No formal randomization will be performed, as it is not considered relevant given the small sample size required.

The **first 10 patients** will receive a Ganirelix dose of 0.025 mg*, and the **next 10 patients** will receive a Ganirelix dose of 0.0625 mg*.

#### ****5.2.2 Blinding****

Neither the participants nor the laboratory personnel performing the hormonal assays will be aware of the dose administered.

### ****5.3 Number of Subjects Required****

A total of **20 patients** will be included, with **10 patients per treatment arm**.

For details regarding the calculation of the required sample size, refer to **Section 11.1 – Calculation of the Number of Subjects Required.**

##

## ****6 ELIGIBILITY CRITERIA AND FOLLOW-UP PROCEDURES****

### ****6.1 Inclusion Criteria****

- Female participant
- Age between **18 and 35 years**
- **Minimum body weight:** 51 kg
- **Body Mass Index (BMI):** below 30 kg/m²
- **Diagnosis of PCOS**, defined by **AMH > 28 pmol/L**, **LH > 8 IU/mL**, and **total testosterone > 0.39 ng/mL**
- No use of **hormonal contraception** or **hormonal treatment** within the past **2 months**
- **Written informed consent** obtained for participation in this **Category 1 clinical trial**
- **Covered by national health insurance**
- Willing and able to comply with all **study procedures** and **study duration**

### ****6.2 Exclusion Criteria****

- Ongoing treatment with **medications affecting metabolism**, such as **metformin**
- **Pregnant woman**
- **Inability to understand** the information sheet
- **Hypersensitivity** to the active substance Ganirelix, to any **structural analogues of gonadotropin-releasing hormone (GnRH)**, to **exogenous peptide hormones**, or to any **excipients**
- **Administrative reasons**, including:
  - Inability to receive adequate informed information
  - Inability to participate in the entire study
  - Lack of **social security coverage**
  - **Refusal to sign** the informed consent form

### ****6.3 Criteria for Study Withdrawal and Early Termination****

- Occurrence of **discomfort** during the 8-hour study period
- **Severe allergic reaction** requiring medical and/or pharmacological intervention
- **Withdrawal requested by the participant**

### ****6.4 Prohibition of Simultaneous Participation and Exclusion Period****

#### ****6.4.1 Simultaneous Participation in Another Study****

No. Participants may not be enrolled in any other clinical trial simultaneously.

#### ****6.4.2 Post-Study Exclusion Period****

None.

#### ****6.4.3 Registration in the Volunteer Database (VRB File)****

Not applicable.

## ****7 CONDUCT OF THE STUDY****

### ****7.1 Feasibility and Experience of the Investigative Team****

The investigators involved in this study are physicians with extensive clinical and research experience in **polycystic ovary syndrome (PCOS)**.
Ganirelix is a compound routinely used by the medical team in **reproductive medicine**.

A **state-registered nurse** and suitable **clinical facilities** are available to conduct all study-related procedures.

### ****7.2 Study Procedures****

This is a **phase 1 pilot study** with **two parallel groups**, evaluating the efficacy of two different doses of Ganirelix in **20 women with PCOS**, defined by **oligo-/anovulation**, **hyperandrogenism**, and **elevated circulating LH levels**.

An **intravenous cannula** will be inserted, and **blood samples will be collected every 10 minutes** for a **4-hour baseline period** starting at 8:00 a.m.
Subsequently, Ganirelix will be administered as a **single subcutaneous injection** at one of two doses:

- **0.025 mg** (n = 10 PCOS patients)
- **0.0625 mg** (n = 10 PCOS patients)

These doses correspond to approximately **one-tenth and one-quarter** of the standard clinical dose used in **IVF protocols** to achieve full LH suppression.

Following Ganirelix administration, blood sampling will continue every 10 minutes for an additional **4-hour period**.

The **primary endpoints** include mean LH levels, as well as LH pulse **frequency and amplitude**, evaluated before and during treatment with Ganirelix.
In healthy women, LH surges occur approximately every 90 minutes during the early follicular phase, whereas **lean women with PCOS** may exhibit **one or more LH pulses per hour**.

The expected outcome is to determine the Ganirelix dose that reduces the **abnormally elevated frequency** of LH pulses by approximately **20–30%**, bringing them closer to normal physiological values.

**Secondary outcomes** include reproductive hormone markers — **serum testosterone, androstenedione, LH, FSH, estradiol, and AMH levels** — measured before treatment and **4 hours post-injection**.

The **pretreatment period** (baseline LH secretion and pulsatility) will be compared to the **4-hour post-antagonist period.**An **LH pulse** will be defined as a **≥20% incremental change** in LH concentration between the nadir and the subsequent peak, as previously described in similar clinical studies (Ref. 25).

The degree of gonadotropin suppression will be calculated as a **percentage of inhibition** relative to the pre-antagonist period:

$$\text{Inhibition (\%)}=\frac{(\text{PRE mean}-\text{nadir})}{\text{PRE mean}}\times100$$

as described previously (Ref. 26).
Hormone nadir levels will be computed using a **moving average**.
For LH, a **6-point moving average** (equivalent to 1 hour of sampling) will be used, as the nadir is expected to occur within the 10-minute sampling intervals.

### ****7.2.1 Patient Selection and Recruitment****

Participants will be recruited during **endocrine gynaecology consultations** at the **Jeanne de Flandre Hospital**.

### ****7.2.2 Information and Informed Consent****

Participants will receive **comprehensive oral and written information** explaining all study procedures.
An **information sheet** will be provided by the investigator or a delegated physician prior to inclusion.

**Written informed consent** must be obtained from each participant **before any protocol-specific procedures**are initiated.
The consent form will be signed by both the **investigator (or delegated physician)** and the **participant**.

The information sheet and consent form will be prepared in **three copies**:

- One copy for the participant
- One retained by the investigator
- One sent to the sponsor in a **sealed envelope**

A **reasonable reflection period (1 week)** will be provided to allow the participant to confirm or decline participation.

### ****7.2.3 Inclusion Visit****

At the inclusion visit, **written informed consent** will be collected, and the **study procedures** will be carried out over a **single study day (8:00 a.m. to 4:00 p.m.)**, scheduled **at the beginning of the menstrual cycle**.

### ****7.2.4 End-of-Study Visit****

Each participant will receive a **follow-up phone call** from **Prof. Sophie Catteau-Jonard 3 to 4 days after study completion**to ensure the absence of adverse effects potentially related to study participation (e.g., redness, skin warmth, itching, urticaria, nasal discharge, rapid or irregular pulse, tongue or throat swelling, sneezing, wheezing, breathing difficulties, dizziness, asthenia, or catheter site discomfort, as well as nausea or headache).

### ****7.3 Study Duration****

- **Expected recruitment period:** 12 months
- **Duration of participation for each subject:** 1 week
- **Total study duration:** 12 months and 1 week

### ****7.4 Study Procedures and Differences from Standard Care****

This is a **clinical research study** involving **one day of hospitalization** for **blood sampling every 10 minutes over 8 hours**.

This procedure does **not correspond to standard medical care**, which typically involves only a **single baseline hormonal assessment**, followed by treatment tailored to the patient’s clinical presentation — such as **ovulation induction** (for infertility), **anti-androgen therapy** (for acne, hirsutism, or alopecia), or **hormonal therapy** to induce withdrawal bleeding (for menstrual irregularities).

# INVESTIGATIONAL MEDICAL PRODUCT

### ****8.1 Name and Description of the Investigational Medicinal Product (IMP)****

**Orgalutran® 0.25 mg** (Ganirelix) will be used at doses **significantly lower** than the recommended therapeutic doses.

For this pilot study:

- **10 participants** will receive an injection corresponding to **one-quarter of the standard dose** (Experimental Group 1), and
- **10 participants** will receive an injection corresponding to **one-tenth of the standard dose** (Experimental Group 2).

The **dilution**of the investigational product will be performed by the **qualified study nurse** prior to administration.

See the **Summary of Product Characteristics (SmPC)** for Ganirelix (Annex 4).

### ****8.2 Labelling of the Investigational Medicinal Product****

The investigational product will be **labelled in accordance with Good Clinical Practice (GCP) and applicable regulatory requirements**, clearly identifying:

- The **name of the investigational product (Ganirelix)**
- The **dose and concentration after dilution**
- The **study identification code**
- The **participant identification number**
- The **administration route (subcutaneous)**
- The **storage conditions**
- The **mention “For clinical trial use only – Not for sale”**

Labelling and documentation will be managed by the **hospital pharmacy** under the supervision of the **principal investigator**.

# BIOLOGICAL ANALYSES

**Routine Clinical Tests and Study-Specific Biological Analyses**

All laboratory analyses will be conducted under the supervision of **Prof. Pascal PIGNY**, Department of **Hormonology, Nutrition, and Oncology**, at the**Institute of Biochemistry and Molecular Biology**, within the **Biology, Pathology, and Genetics Division** of **Lille University Hospital (CHU de Lille).**

| **Parameters to be Analyzed** | **Baseline (T0)** | **Intermediate Time Points (every 10 min for 8 h)** | **Final Time Point (T0 + 8 h)** | **Use (Clinical/Research)** | **Location & Responsible Unit** |
| --- | --- | --- | --- | --- | --- |
| LH | — | 1 dry tube (1.5 mL) at each time point | — | Research | CBP Biochemistry Laboratory |
| LH, FSH, Estradiol, Total Testosterone | 1 dry tube (5 mL) | — | 1 dry tube (5 mL) | Research | CBP Biochemistry Laboratory |
| AMH | 1 dry tube (5 mL) | — | 1 dry tube (5 mL) | Research | CBP Biochemistry Laboratory |
| Androstenedione | 1 dry tube (5 mL) | — | 1 dry tube (5 mL) | Research | CBP Biochemistry Laboratory |
| **Total** | **3 dry tubes (5 mL)** | **49 dry tubes (1.5 mL)** | **3 dry tubes (5 mL)** | **Research** | **CBP Biochemistry Laboratory** |

### ****Sample Storage and Handling****

Samples will be stored for **3 months** at the **CBP Laboratory**, following the same procedure as for routine hormonal assays.

- **AMH samples** will be stored for **1 year**.
- **Other hormonal assays** will be stored for **1 week**.

All samples are collected **exclusively for the purpose of this study** and will be **destroyed after analysis**, with **no biological collection or biobank** established.

### ****Total Blood Volume Collected****

| **Type of Analysis** | **Volume (mL)** |
| --- | --- |
| Biology | 103.5 mL |
| Pharmacokinetics / Efficacy | 0 mL |
| Genotyping | 0 mL |
| **Total** | **103.5 mL** |

## ****BIOLOGICAL SAMPLE COLLECTION****

**Not applicable.**
No biological collection or long-term biobank will be constituted as part of this study.

# SAFETY EVALUATION

### ****10.1 Definitions****

**Adverse Event (AE):**
Any untoward medical occurrence in a participant enrolled in a clinical study involving human subjects, whether or not it is related to the research or to the investigational product.

**Adverse Reaction (AR):**
Any adverse event considered to be related to the research or to the investigational product.

**Serious Adverse Event or Serious Adverse Reaction (SAE/SAR):**
Any event or reaction that:

- results in death,
- is life-threatening,
- requires inpatient hospitalization or prolongation of existing hospitalization,
- results in persistent or significant disability/incapacity,
- results in a congenital anomaly or birth defect, or
- is considered medically significant by the investigator.

Certain hospitalizations do not fall under the criterion of “seriousness” when they are:

- admissions for social or administrative reasons,
- hospitalizations predefined in the protocol,
- hospitalizations for medical or surgical treatment scheduled prior to the study,
- or day-hospital admissions.

**Unexpected Adverse Reaction (UAR):**
Any adverse reaction whose nature, severity, or outcome is not consistent with the information available in the product documentation, procedures, or methods used in the study.

**New Safety Information (“Fait nouveau”):**
Any new data that may lead to a reassessment of the benefit-risk balance of the study or of the investigational product, require modifications in the use of the product, in the conduct of the study, in study-related documentation, or justify suspension, interruption, or modification of the study protocol or similar studies.

### ****10.2 Description of Safety Evaluation Parameters and Protocol-Related Risks****

**Risks Related to Treatment:**
Possible allergy to Ganirelix manifested by redness, skin warmth, itching, urticaria, nasal discharge, rapid or irregular pulse, swelling of the tongue and throat, sneezing, wheezing, severe breathing difficulties, or dizziness.
Special caution is required in women presenting with signs or symptoms of an active allergic condition or with a known history of allergic predisposition. Treatment with Ganirelix is **not recommended** for women with severe allergic disorders.

**Other risks described in the Summary of Product Characteristics (SmPC) for Orgalutran:**

- Ovarian hyperstimulation syndrome (OHSS): lower abdominal pain with nausea or vomiting, decreased urine output, weight gain, or breathing difficulties (not applicable in this study context);
- Skin irritation at the injection site: redness, itching, swelling;
- Nausea;
- Headache.

**Risks Related to Blood Sampling:**
Blood collection may cause very slight discomfort due to frequent sampling, and mild bruising may occur at the site for several days following the blood draws.

### ****10.3 Procedures for Recording and Reporting Adverse Events****

#### ****Responsibilities of the Investigator****

**Collection of Adverse Events:**
All adverse events will be recorded on the adverse event report forms included in the case report form (CRF). Each adverse event will be documented individually.

**Severity grading** will be determined as follows:

- Mild (Grade 1): no interference with daily activities;
- Moderate (Grade 2): moderate interference with daily activities but acceptable;
- Severe (Grade 3): marked interference with daily activities, unacceptable;
- Life-threatening (Grade 4);
- Death (Grade 5).

All adverse events must be **graded and assessed** for **causality and outcome**.

#### ****Reporting of Serious Adverse Events (SAEs)****

The investigator must notify the sponsor **immediately upon awareness** of any **serious adverse event** occurring during the study period, except for those explicitly listed in the protocol as **not requiring notification**.

Each SAE must be documented using the **“Serious Adverse Event” reporting form** provided in the CRF and transmitted to the sponsor’s **Clinical Trials Vigilance Unit (Direction de la Recherche et de l’Innovation, CHU Lille)** by:

- **Email:** vigilance.essaiscliniques@chru-lille.fr

For each SAE, the investigator must provide detailed documentation including:

- a clear and complete description of the event (preferably as a medical diagnosis),
- severity, start and end dates, outcome,
- assessment of the causal relationship between the SAE and:
  - the investigational product and/or
  - any concomitant treatment.

Follow-up of all adverse events will be ensured by the investigator until resolution or stabilization.

Whenever possible, the investigator must attach anonymized supporting documents:

- hospitalization or hospital extension reports,
- relevant laboratory or imaging results,
- any other documentation deemed useful.

#### ****Reporting Period for Serious Adverse Events****

All SAEs must be reported if they occur:

- from the **date of signature of the informed consent**,
- throughout the participant’s study follow-up period,
- and for a **minimum of 7 days** after completion of the study procedures.

There is **no time limitation** for reporting SAEs considered likely to be related to the study or to the investigational product (e.g., delayed effects such as cancer or congenital anomalies).

#### ****Reporting of Pregnancies****

Pregnancy is **not considered a serious adverse event**, but any pregnancy occurring during the trial must be **reported immediately** using the **standard pregnancy notification form** to the sponsor.

The sponsor will ensure appropriate follow-up if deemed necessary.
The investigator must follow the participant until the **end of the pregnancy or its termination** and report the outcome using the **standard pregnancy outcome form**.

If the pregnancy outcome meets SAE criteria (e.g., spontaneous abortion with hospitalization, fetal death, congenital anomaly), the **SAE reporting procedure** must be followed.

### ****10.4 Responsibilities of the Sponsor****

#### ****Reporting of Serious and Unexpected Adverse Reactions (SUSARs)****

For each SAE, the sponsor will assess **severity**, **causality**, and **unexpectedness** with respect to the investigational product and/or concomitant treatments.

The sponsor must report all **suspected unexpected serious adverse reactions (SUSARs)** to the **ANSM** (French Medicines Agency) and the **Ethics Committee (CPP)**:

- **Immediately** (without delay) for any **fatal or life-threatening** SUSAR;
- **Within 15 days** of awareness for all **other SUSARs**.

Follow-up reports providing relevant additional safety information will be sent:

- **Within 8 days** of the initial report for fatal or life-threatening SUSARs;
- **Within 8 days** following the 15-day initial report for other SUSARs.

#### ****Reporting of New Safety Information****

In the event of **new safety information**, the sponsor will immediately notify the **CPP** and **ANSM** by email, as soon as such information becomes known, along with any measures taken in response.

#### ****Annual Safety Report****

Once per year for the duration of the study (or upon request), the sponsor will submit to the **ANSM** and the **CPP** an **annual safety report**.
This report will include a **comprehensive analysis** of the study’s safety profile, incorporating all newly available safety data.
It will include **summary tables** of all **serious adverse events and reactions** that occurred during the clinical trial.

### ****10.5 Independent Data Safety Monitoring Committee****

Given the existing knowledge regarding Ganirelix and the **low doses** used in this study population, the establishment of an **Independent Data Safety Monitoring Committee (DSMC)** is **not considered necessary**.

# DATA MANAGEMENT

The individual data collected during the study will first be recorded on a source document, then entered electronically to create a computerized database.
All data are confidential in accordance with the French Data Protection Act of January 6, 1978, and the General Data Protection Regulation (GDPR).

Data processing will be carried out in compliance with the Reference Methodology MR-001 established by the CNIL (Commission Nationale de l’Informatique et des Libertés), within the Statistical, Economic Evaluation and Data Management Unit (SEED), under the supervision of Ms. Hajar Chouiki at Lille University Hospital (CHU de Lille).

The data will be entered into a secure electronic database using the software Ennov Clinical, which is certified for data management in clinical trials and compliant with FDA (Food and Drug Administration) recommendations.
All data will be hosted on a secured server belonging to CHU de Lille.

This software enables the creation of an electronic case report form (eCRF) and the implementation of data validation and consistency rules, allowing automatic query generation during data entry.
Before database lock, a data manager will perform data monitoring through Ennov Clinical, based on predefined consistency rules established in collaboration with the study’s principal investigator.

Access to the data will be restricted to individuals directly involved in the study.
Data may be modified only by an investigator-physician participating in the study or by a delegated collaborator explicitly authorized to do so.

All data related to this study will be archived for a minimum of fifteen (15) years from the end of the research or its early termination, without prejudice to the applicable legal and regulatory requirements in force.

# STATISTICAL ANALYSES

**General Principles**
All statistical analyses will be performed independently by the Department of Biostatistics, University of Lille, under the supervision of Mr. Julien Labreuche. Analyses will be conducted using 10.0.2 GraphPad Prism software (GraphPad; San Diego, CA, USA).

All analyses will be performed on the per-protocol population, unless otherwise specified. The level of statistical significance will be set at α = 0.05 (two-sided). No adjustment for multiple testing will be applied, and all secondary analyses will be considered exploratory. Results will be presented as effect size estimates with corresponding 95% confidence intervals (95% CI).

**Description of Data**
Baseline demographic and clinical characteristics will be described by treatment group.
Quantitative variables will be summarized as mean (standard deviation or standard error). The normality of data distributions will be evaluated using the Shapiro–Wilk test.

For normally-distributed data, data will be compared using unpaired two-sided Student’s *t*-test. For non-normally distributed values, Mann-Whitney U test will be used.

**Primary Endpoint Analysis**
The primary endpoint is the reduction in LH pulse frequency during the 4-hour period following Ganirelix administration.
Analysis will include:
- Determination of LH pulse frequency using the method described above (1.1.2).

**Secondary Endpoint Analyses**
- LH variation (mean, basal and total LH) before and after Ganirelix injection will be assessed.
- Androgen production variation will be evaluated as the difference in total testosterone and androstenedione levels between H8 and H0 (4 hours post- vs. pre-injection).
- FSH, estradiol, and AMH concentrations will be compared between H0 and H8 to assess hormonal changes after treatment.

**Handling of Missing Data**
Given the short study duration and intensive monitoring, missing data are expected to be minimal. Any missing measurements will be described, and no imputation is planned. Analyses will be based on available data only.

**Statistical Software**
All statistical analyses will be performed using:
10.0.2 GraphPad Prism software (GraphPad; San Diego, CA, USA)

**Justification for the Absence of a Power Calculation**As this is an **exploratory study** no formal sample size calculation or statistical power analysis has been performed. The main objective of this research is to generate preliminary physiological and pharmacodynamic data on the effect of low-dose Ganirelix on LH pulsatility in women with PCOS, rather than to formally test a hypothesis.
Given the mechanistic and proof-of-concept nature of the study, the selected sample size of 20 participants is deemed appropriate to:
- Evaluate feasibility and study logistics;
- Provide initial estimates of variability in LH pulse frequency and amplitude under low-dose GnRH antagonist treatment;
- Support the design of a future adequately powered confirmatory trial.
This approach is consistent with international regulatory guidance for early-phase exploratory studies (ICH E9, EMA guidelines on exploratory trials), which emphasize estimation over formal hypothesis testing in pilot settings.

## 13 QUALITY CONTROL AND ASSURANCE

**Conduct of the study**

A start-up meeting with the principal investigator will be held before the beginning of the study to review Good Clinical Practice (GCP) requirements, the organization of the trial, and the planned monitoring procedures.
The investigator will inform the sponsor in real time of all inclusions made.

Medical observations will be kept in the patient’s medical record, and all study-related data will be entered into the designated case report forms (CRFs) in accordance with Good Clinical Practice. These forms will document each step of the participant’s management according to the study protocol.
Any protocol deviation will be documented along with its justification.
Data collection must be exhaustive and will be regularly verified by a Clinical Research Associate (CRA), according to the procedures defined in the protocol (if applicable).

**Monitoring of the study**

Monitoring will be conducted according to the monitoring plan approved before the start of the study or triggered by a specific request from the sponsor’s CRA. The frequency and extent of monitoring activities will depend on the number of inclusions and study progress.

During on-site monitoring visits, CRAs must be able to access:

- The patients’ case report forms (CRFs)
- The medical and nursing records of enrolled participants
- The investigator site file

Monitoring will verify at least the following points:

- The existence of participants and the presence of signed informed consent forms
- Compliance with inclusion criteria
- Verification of the primary endpoint
- Recording and reporting of serious adverse events (SAEs)
- Occurrence of any new information requiring the submission of a protocol amendment
- Management and monitoring of investigational product handling by the pharmacy

**Closure of the study**

At the end of the study, closure procedures will be implemented, including the organization and filing of all source documents and data. Once the final analysis has been completed and validated, all study documentation and data will be sealed and archived according to specific procedures in secure facilities.

## 14 ETHICAL AND LEGAL CONSIDERATIONS

The study will be conducted in accordance with the approved protocol, the French Public Health Code, the principles of Good Clinical Practice (GCP) of the European Union, and all applicable regulatory requirements.
The trial will be registered in the public database ClinicalTrials.gov.

### Ethics Committee and Competent Authority

Authorization from the Competent Authority and opinion from the Ethics Committee

Before the beginning of the research, the sponsor will submit an authorization request to the ANSM (Agence Nationale de Sécurité du Médicament et des Produits de Santé) and will obtain a favorable opinion from the Ethics Committee (Comité de Protection des Personnes, CPP) in accordance with Article L1121-4 of the French Public Health Code.

### Protocol Amendments

Only the sponsor is authorized to modify the protocol, in consultation with the coordinating investigator.

A substantial amendment refers to any modification that significantly impacts any aspect of the research, including the protection and safety of participants, the validity of the study, the quality and safety of the investigational product, the interpretation of scientific documentation supporting the study, or the study’s conduct.
A request for approval of a substantial amendment will be submitted by the sponsor to the ANSM, the CPP, or both, depending on the nature of the change, for authorization and/or opinion. Once authorization and/or a favorable opinion has been received, the amended version of the protocol will be distributed by the sponsor to all investigators.

A non-substantial amendment refers to a minor change or clarification with no impact on the conduct of the trial. Such modifications will not be submitted to the authorities but will be agreed upon between the sponsor and the investigator and clearly documented in the study’s follow-up file.

### Participant Information and Consent

In accordance with current regulations, participants must receive complete and understandable information through a Participant Information Sheet specifically prepared for the study and approved by the Ethics Committee. This information will be provided and explained by the investigator.
The investigator must ensure that participants are fully informed about potential risks and constraints associated with participation in the study.

Participants will be given sufficient time to ask questions and to make an informed decision.
They must then sign the Informed Consent Form in the presence of the investigator who presented the study, and the document must be dated on the day of signing. One copy will be given to the participant, one retained by the investigator, and one filed in the participant’s medical record.

The investigator must ensure compliance with all inclusion and exclusion criteria prior to enrollment. No study-specific procedures may take place before the participant has been fully informed and has provided written consent.
Participants may withdraw their consent at any time, and any new information relevant to their safety will be promptly communicated to them.

### Data Protection and CNIL Compliance

In accordance with the Reference Methodology MR-001, data processing will be carried out under the confidentiality conditions defined by the amended French Data Protection Act of January 6, 1978, and in compliance with the European General Data Protection Regulation (GDPR).

The data controller is Lille University Hospital (CHU de Lille), acting as the sponsor, whose contact details are:
Direction de la Recherche et de l’Innovation – Maison Régionale de la Recherche Clinique, Hospitalière et Universitaire,
6 rue du Professeur Laguesse – CS 70001 – 59037 Lille Cedex, France.

In accordance with the GDPR, data from this study will be processed for the purpose of scientific research (Article 6) within the framework of a mission of public interest (Article 9) for which the sponsor is responsible.
The data will be stored on the secure CHU Lille network and will be pseudonymized.

In compliance with Article L1121-1-1 of the French Public Health Code and Articles 17.3.c and 17.3.d of the GDPR, participants have the right to request the erasure of their data and to object to its processing. They will be informed that data collected prior to withdrawal of consent may not be deleted and may continue to be processed under the conditions defined for the research.
Participants also have the right to request restriction of processing in accordance with Article 18 of the GDPR, and to lodge a complaint with the supervisory authority (CNIL in France).
In accordance with Article 13 of the GDPR, when personal data are collected directly from the participant, the data controller must provide all relevant information required by this article at the time of collection.

For the purpose of the research, personal data will be processed to allow analysis of study results in relation to the study objectives.
To this end, medical and other data concerning participants may be transmitted to the sponsor or to third parties or organizations acting on behalf of the sponsor, in France or abroad, including outside the European Union, provided that the destination country ensures an adequate level of data protection as recognized by French authorities.
These data will be identified by a unique code number and participant initials.
Under conditions ensuring confidentiality, data may also be transmitted to French and foreign health authorities.

All study-related data will be archived for a minimum of fifteen years from the end or early termination of the study, in accordance with applicable legislative and regulatory provisions.

## 15 FUNDING AND INSURANCE

### Funding

The study is funded by the **Horizon-ERC-POC grant (ERC-2022-POC2 to P.G., grant agreement n° 101111874/GRASP)**

***Insurance***

The sponsor will obtain an insurance policy covering its civil liability as well as that of all study personnel, in accordance with Article L1121-10 of the French Public Health Code.
The study may not begin until this insurance coverage has been secured.

### PUBLICATION AND DISSEMINATION OF RESULTS

#### Decision on Publication

The final study report will be written by the coordinating investigator, Professor Sophie Catteau-Jonard, in collaboration with her research team.

In accordance with Article R5121-13 of the French Public Health Code, no written or oral communication about this trial may be made without the joint agreement of both the investigator and the sponsor.
All publications must state that Lille University Hospital (CHU de Lille) is the sponsor of the study.
In any case, CHU de Lille, as the sponsor, retains control over the first publication.
The investigator will provide the sponsor with a copy of any intended publication or presentation.

#### Publication Rules

Professor Sophie Catteau-Jonard and Paolo Giacobini will be included as first or last authors on all resulting publications.
The sponsor retains exclusive ownership of all study results and related data.
These results and all research data may not be transmitted to any third party without prior negotiation and formal agreement with the Directorate for Research and Innovation.
Any such request must be communicated as early as possible to the Legal Affairs Office of the Directorate for Research and Innovation.

#### Publication of Ancillary Studies

Not applicable.

#### Research Valorization

The study results and all related data may not be disclosed or transferred to any third party without prior negotiation and authorization from the Directorate for Research and Innovation.
Any such request must be submitted as soon as possible to the Legal Affairs Office of the Directorate for Research and Innovation.

Annexe 1 : Bibliography

1. Walters, K.A.*, et al.* New Perspectives on the Pathogenesis of PCOS: Neuroendocrine Origins. *Trends in endocrinology and metabolism: TEM* **29**, 841-852 (2018).

2. Escobar-Morreale, H.F. Polycystic ovary syndrome: definition, aetiology, diagnosis and treatment. *Nature reviews. Endocrinology* **14**, 270-284 (2018).

3. Stener-Victorin, E. & Deng, Q. Epigenetic inheritance of polycystic ovary syndrome - challenges and opportunities for treatment. *Nature reviews. Endocrinology* **17**, 521-533 (2021).

4. Azziz, R., Marin, C., Hoq, L., Badamgarav, E. & Song, P. Health care-related economic burden of the polycystic ovary syndrome during the reproductive life span. *The Journal of clinical endocrinology and metabolism* **90**, 4650-4658 (2005).

5. Teede, H.J.*, et al.* Recommendations from the international evidence-based guideline for the assessment and management of polycystic ovary syndrome. *Human reproduction* **33**, 1602-1618 (2018).

6. Livadas, S.*, et al.* Prevalence and impact of hyperandrogenemia in 1,218 women with polycystic ovary syndrome. *Endocrine* **47**, 631-638 (2014).

7. Morales, A.J.*, et al.* Insulin, somatotropic, and luteinizing hormone axes in lean and obese women with polycystic ovary syndrome: common and distinct features. *The Journal of clinical endocrinology and metabolism* **81**, 2854-2864 (1996).

8. Taylor, A.E.*, et al.* Determinants of abnormal gonadotropin secretion in clinically defined women with polycystic ovary syndrome. *The Journal of clinical endocrinology and metabolism* **82**, 2248-2256 (1997).

9. Ehrmann, D.A. Polycystic ovary syndrome. *The New England journal of medicine* **352**, 1223-1236 (2005).

10. Goodarzi, M.O., Dumesic, D.A., Chazenbalk, G. & Azziz, R. Polycystic ovary syndrome: etiology, pathogenesis and diagnosis. *Nature reviews. Endocrinology* **7**, 219-231 (2011).

11. McCartney, C.R. & Campbell, R.E. Abnormal GnRH Pulsatility in Polycystic Ovary Syndrome: Recent Insights. *Curr Opin Endocr Metab Res* **12**, 78-84 (2020).

12. Cimino, I.*, et al.* Novel role for anti-Mullerian hormone in the regulation of GnRH neuron excitability and hormone secretion. *Nature communications* **7**, 10055 (2016).

13. Silva, M.S.B. & Giacobini, P. New insights into anti-Mullerian hormone role in the hypothalamic-pituitary-gonadal axis and neuroendocrine development. *Cellular and molecular life sciences : CMLS* **78**, 1-16 (2021).

14. Tata, B.*, et al.* Elevated prenatal anti-Mullerian hormone reprograms the fetus and induces polycystic ovary syndrome in adulthood. *Nat Med* (2018).

15. Vigier, B., Picard, J.Y., Tran, D., Legeai, L. & Josso, N. Production of anti-Mullerian hormone: another homology between Sertoli and granulosa cells. *Endocrinology* **114**, 1315-1320 (1984).

16. Durlinger, A.L.*, et al.* Control of primordial follicle recruitment by anti-Mullerian hormone in the mouse ovary. *Endocrinology* **140**, 5789-5796 (1999).

17. Cook, C.L., Siow, Y., Brenner, A.G. & Fallat, M.E. Relationship between serum mullerian-inhibiting substance and other reproductive hormones in untreated women with polycystic ovary syndrome and normal women. *Fertility and sterility* **77**, 141-146 (2002).

18. Pigny, P., Jonard, S., Robert, Y. & Dewailly, D. Serum anti-Mullerian hormone as a surrogate for antral follicle count for definition of the polycystic ovary syndrome. *The Journal of clinical endocrinology and metabolism* **91**, 941-945 (2006).

19. Pellatt, L.*, et al.* Granulosa cell production of anti-Mullerian hormone is increased in polycystic ovaries. *The Journal of clinical endocrinology and metabolism* **92**, 240-245 (2007).

20. Pigny, P.*, et al.* Elevated serum level of anti-mullerian hormone in patients with polycystic ovary syndrome: relationship to the ovarian follicle excess and to the follicular arrest. *The Journal of clinical endocrinology and metabolism* **88**, 5957-5962 (2003).

21. Piltonen, T.T.*, et al.* Circulating antimullerian hormone and steroid hormone levels remain high in pregnant women with polycystic ovary syndrome at term. *Fertility and sterility* **111**, 588-596 e581 (2019).

22. Mimouni, N.E.H.*, et al.* Polycystic ovary syndrome is transmitted via a transgenerational epigenetic process. *Cell metabolism* **33**, 513-530 e518 (2021).

23. Maliqueo, M.*, et al.* Placental steroidogenesis in pregnant women with polycystic ovary syndrome. *European journal of obstetrics, gynecology, and reproductive biology* **166**, 151-155 (2013).

24. Steingold, K.*, et al.* Clinical and hormonal effects of chronic gonadotropin-releasing hormone agonist treatment in polycystic ovarian disease. *The Journal of clinical endocrinology and metabolism* **65**, 773-778 (1987).

25. Rebar, R., Judd, H.L., Yen, S.S., Rakoff, J., Vandenberg, G., and Naftolin, F. (1976). Characterization of the inappropriate gonadotropin secretion in polycystic ovary syndrome. The Journal of clinical investigation *57*, 1320-1329. 10.1172/JCI108400

26. Hayes, F.J., Taylor, A.E., Martin, K.A., and Hall, J.E. (1998). Use of a gonadotropin-releasing hormone antagonist as a physiologic probe in polycystic ovary syndrome: assessment of neuroendocrine and androgen dynamics. J Clin Endocrinol Metab *83*, 2343-2349. 10.1210/jcem.83.7.4925.

Annexe 2 : Volume of Blood Collected in Relation to Participant Body Weight

| **Maximum Allowable Blood Volume (Clinical Care + Research) According to Body Weight** | | | | | |
| --- | --- | --- | --- | --- | --- |
| **Body Weight (kg)** | **Total blood volume mL** | **Maximum Volume per Single Session (mL) (= 2.5 % of the total blood volume)** | **Maximum Total Blood Volume (Clinical Care + Research) per 30-Day Period (mL)** | **Minimum Hemoglobin Level Required at the Time of Blood Sampling** | **Minimum Hemoglobin Level Required at the Time of Blood Sampling for Participants with Respiratory or Cardiovascular Disease** |
| 1 | 100 | 2.5 | 5 | 7.0 | 9.0-10.0 |
| 2 | 200 | 5 | 10 | 7.0 | 9.0-10.0 |
| 3 | 240 | 6 | 12 | 7.0 | 9.0-10.0 |
| 4 | 320 | 8 | 16 | 7.0 | 9.0-10.0 |
| 5 | 400 | 10 | 20 | 7.0 | 9.0-10.0 |
| 6 | 480 | 12 | 24 | 7.0 | 9.0-10.0 |
| 7 | 560 | 14 | 28 | 7.0 | 9.0-10.0 |
| 8 | 640 | 16 | 32 | 7.0 | 9.0-10.0 |
| 9 | 720 | 18 | 36 | 7.0 | 9.0-10.0 |
| 10 | 800 | 20 | 40 | 7.0 | 9.0-10.0 |
| 11-15 | 880-1 200 | 22-30 | 44-60 | 7.0 | 9.0-10.0 |
| 16-20 | 1 280-1 600 | 32-40 | 64-80 | 7.0 | 9.0-10.0 |
| 21-25 | 1 680-2 000 | 42-50 | 64-100 | 7.0 | 9.0-10.0 |
| 26-30 | 2 080-2 400 | 52-60 | 104-120 | 7.0 | 9.0-10.0 |
| 31-35 | 2 480-2 800 | 62-70 | 124-140 | 7.0 | 9.0-10.0 |
| 36-40 | 2 880-3 200 | 72-80 | 144-160 | 7.0 | 9.0-10.0 |
| 41-45 | 3 280-3 600 | 82-90 | 164-180 | 7.0 | 9.0-10.0 |
| 46-50 | 3 680-4 000 | 92-100 | 184-200 | 7.0 | 9.0-10.0 |
| 51-55 | 4 080-4 400 | 102-110 | 204-220 | 7.0 | 9.0-10.0 |
| 56-60 | 4 480-4 800 | 112-120 | 224-240 | 7.0 | 9.0-10.0 |
| 61-65 | 4 880-5 200 | 122-130 | 244-260 | 7.0 | 9.0-10.0 |
| 68-70 | 5 280-5 600 | 132-140 | 264-280 | 7.0 | 9.0-10.0 |
| 71-75 | 5 680-6 000 | 142-150 | 284-300 | 7.0 | 9.0-10.0 |
| 76-80 | 6 080-6 400 | 152-160 | 304-360 | 7.0 | 9.0-10.0 |
| 81-85 | 6 480-6 800 | 162-170 | 324-340 | 7.0 | 9.0-10.0 |
| 86-90 | 6 880-7 200 | 172-180 | 344-360 | 7.0 | 9.0-10.0 |
| 91-95 | 7 280-7 600 | 182-190 | 364-380 | 7.0 | 9.0-10.0 |
| > 96 | 7 680-8 000 | 192-200 | 384-400 | 7.0 | 9.0-10.0 |
|  | | | | | |

Annexe 3 : Biological Sample Flow and Management

| **Parameters to be Analyzed** | **Matrix** | **Baseline Sampling (T0)** | **Intermediate Time Points (every 10 min for 8 h)** | **Final Time Point (T0 + 8 h)** | **Use (Clinical Care or Research)** | **Transport Conditions / Delivery Time (if applicable)** | **Frequency of Shipment or Receipt** | **Location and Responsible Laboratory** | **Final Disposition of Samples** |
| --- | --- | --- | --- | --- | --- | --- | --- | --- | --- |
| LH | Blood | – | 1 dry tube (1.5 mL) | – | Research | Immediate transport (room temperature) | As participants are enrolled | CBP Biochemistry Laboratory | Destruction after analysis |
| LH, FSH, E2, Total Testosterone | Blood | 1 dry tube (5 mL) | – | 1 dry tube (5 mL) | Research | Immediate transport (room temperature) | As participants are enrolled | CBP Biochemistry Laboratory | Destruction after analysis |
| AMH | Blood | 1 dry tube (5 mL) | – | 1 dry tube (5 mL) | Research | Immediate transport (room temperature) | As participants are enrolled | CBP Biochemistry Laboratory | Destruction after analysis |
| Androstenedione | Blood | 1 dry tube (5 mL) | – | 1 dry tube (5 mL) | Research | Immediate transport (room temperature) | As participants are enrolled | CBP Biochemistry Laboratory | Destruction after analysis |
| **TOTAL** | Blood | 3 dry tubes (5 mL) | 49 dry tubes (1.5 mL) | 3 dry tubes (5 mL) | Research | Immediate transport (room temperature) | As participants are enrolled | CBP Biochemistry Laboratory | Destruction after analysis |

**Notes:**

- TAL: immediate transport to the analysis laboratory.
- All samples are collected exclusively for research purposes.
- Samples will be destroyed after completion of analyses, with no biological collection or biobank constituted.

### Annex 4: Summary of Product Characteristics (SmPC) for Ganirelix*

**Source:** Vidal Database
**Section:** Medicinal Products
**Last Update:** July 29, 2021

**ORGALUTRAN^®^
ganirélix**

[Formes et présentations](http://hoptimal.chrul.net/showProduct.html?productId=18619#forme)|[Composition](http://hoptimal.chrul.net/showProduct.html?productId=18619#compo)|[Indications](http://hoptimal.chrul.net/showProduct.html?productId=18619#indic)|[Posologie et mode d'administration](http://hoptimal.chrul.net/showProduct.html?productId=18619#posol)|[Contre-indications](http://hoptimal.chrul.net/showProduct.html?productId=18619#contr)|[Mises en garde et précautions d'emploi](http://hoptimal.chrul.net/showProduct.html?productId=18619#mises)|[Interactions](http://hoptimal.chrul.net/showProduct.html?productId=18619#inter)|[Fertilité/grossesse/allaitement](http://hoptimal.chrul.net/showProduct.html?productId=18619#gross)|[Conduite et utilisation de machines](http://hoptimal.chrul.net/showProduct.html?productId=18619#machi)|[Effets indésirables](http://hoptimal.chrul.net/showProduct.html?productId=18619#effet)|[Surdosage](http://hoptimal.chrul.net/showProduct.html?productId=18619#surdo)|[Pharmacodynamie](http://hoptimal.chrul.net/showProduct.html?productId=18619#phard)|[Pharmacocinétique](http://hoptimal.chrul.net/showProduct.html?productId=18619#pharc)|[Sécurité préclinique](http://hoptimal.chrul.net/showProduct.html?productId=18619#secur)|[Incompatibilités](http://hoptimal.chrul.net/showProduct.html?productId=18619#incom)|[Modalités de conservation](http://hoptimal.chrul.net/showProduct.html?productId=18619#condi)|[Modalités manipulation/élimination](http://hoptimal.chrul.net/showProduct.html?productId=18619#modal)|[Prescription/délivrance/prise en charge](http://hoptimal.chrul.net/showProduct.html?productId=18619#rensadm)

#### Pharmaceutical Form and Presentation

Subcutaneous injectable solution, 0.25 mg/0.5 mL (aqueous, sterile, ready-to-use, clear and colorless):
Single-use prefilled syringe of 0.5 mL with a needle fitted with a natural dry rubber/latex cap (in contact with the needle; see Warnings and Precautions), available in boxes of 1 and 5 syringes.

#### Composition

**Per syringe:**

- Ganirelix* (INN) ............................................. 0.25 mg
- Excipients: acetic acid, mannitol, water for injections.
  The pH may be adjusted with sodium hydroxide and acetic acid.

**Excipient with known effect:** This medicine contains less than 1 mmol sodium (23 mg) per injection, i.e., it is essentially “sodium-free.”

Ganirelix is a synthetic decapeptide with high antagonistic activity toward natural gonadotropin-releasing hormone (GnRH). Amino acids in positions 1, 2, 3, 6, 8, and 10 of the natural GnRH decapeptide are substituted, yielding [N-Ac-D-Nal(2)1, D-pClPhe2, D-Pal(3)3, D-hArg(Et2)6, L-hArg(Et2)8, D-Ala10]-GnRH, molecular weight 1570.4.

#### Therapeutic Indications

Orgalutran is indicated for the prevention of premature luteinizing hormone (LH) surges in women undergoing controlled ovarian hyperstimulation (COH) in assisted reproductive technology (ART) procedures.
In clinical studies, Orgalutran was used in combination with recombinant follicle-stimulating hormone (FSH) or corifollitropin alfa, a long-acting follicular stimulant.

#### Dosage and Administration

Orgalutran should be prescribed only by specialists experienced in fertility treatment.

**Dosage:**
Orgalutran (0.25 mg) is administered subcutaneously once daily to prevent premature LH surges in women undergoing COH. Stimulation with FSH or corifollitropin alfa begins on day 2 or 3 of the menstrual cycle. Orgalutran treatment begins on day 5 or 6 of stimulation, depending on ovarian response (number and size of growing follicles and/or circulating estradiol levels).

Daily Orgalutran injections continue until sufficient follicular development is achieved. Follicular maturation is then induced by human chorionic gonadotropin (hCG).

Due to its half-life, the interval between two injections or between the last injection and hCG administration must not exceed 30 hours to prevent a premature LH surge.

**Route of administration:**
Subcutaneous injection, preferably in the thigh. Injection sites should vary to avoid lipodystrophy. Patients may self-administer after adequate training.

#### Contraindications

- Hypersensitivity to ganirelix, GnRH, any GnRH analogs, or any excipients listed above.
- Moderate to severe hepatic or renal impairment.
- Pregnancy or breastfeeding.

#### Warnings and Precautions

- **Hypersensitivity reactions:** Rare generalized or local allergic reactions have been reported, including anaphylaxis, angioedema, and urticaria. Treatment should be discontinued if hypersensitivity occurs.
- **Latex allergy:** The needle cap contains natural dry rubber/latex which may cause allergic reactions.
- **Ovarian hyperstimulation syndrome (OHSS):** May occur during or after gonadotropin stimulation.
- **Ectopic pregnancy:** Risk is higher in ART patients with tubal abnormalities.
- **Congenital malformations:** Slightly higher incidence in ART compared to natural conception, likely due to parental factors.
- **Weight considerations:** Safety and efficacy not established in women <50 kg or >90 kg.

#### Interactions

No formal interaction studies have been conducted. Interactions with commonly used drugs, including histamine-releasing agents, cannot be excluded.

#### Fertility, Pregnancy, and Lactation

- **Pregnancy:** Contraindicated.
- **Lactation:** Contraindicated. Unknown if excreted in breast milk.
- **Fertility:** Used to prevent premature LH surges during COH for ART procedures.

#### Adverse Effects

**Very common (≥1/10):** Local injection site reactions (mainly redness ± swelling).
**Common (≥1/100, <1/10):** Malaise.
**Uncommon (≥1/1,000, <1/100):** Headache, nausea.
**Very rare (<1/10,000):** Hypersensitivity reactions (rash, facial swelling, dyspnea, anaphylaxis, angioedema, urticaria).

#### Overdose

Prolonged duration of action may occur. No systemic toxicity reported at doses up to 12 mg SC.

#### Pharmacodynamics

**ATC Code:** H01CC01
**Class:** Hypothalamic and pituitary hormones and analogs, GnRH antagonists.
Ganirelix binds competitively to GnRH receptors in the pituitary, leading to rapid, reversible suppression of LH and FSH without the initial stimulation seen with GnRH agonists.

#### Pharmacokinetics

- **Absorption:** Cmax ≈ 15 ng/mL in 1–2 h after SC administration. Bioavailability ≈ 91%.
- **Half-life:** ≈ 13 h; clearance ≈ 2.4 L/h.
- **Elimination:** 75% feces, 22% urine.

#### Storage and Handling

- Shelf life: 3 years.
- Do not freeze.
- Store in the original packaging, protected from light.
- Use only clear, particle-free solutions. Dispose of any unused product per regulations.

#### Regulatory and Administrative Information

- **Prescription class:** List I – restricted to gynecology, obstetrics, endocrinology, or metabolism specialists.
- **Marketing Authorization:**
  - EU/1/00/130/001 (1 syringe)
  - EU/1/00/130/002 (5 syringes)
  - MA holder: N.V. Organon, Oss, Netherlands.
  - French distributor: Organon France, 106 Boulevard Haussmann, 75008 Paris.
- **Prices:** €22.89 (box of 1), €113.12 (box of 5).
- **Reimbursement:** 100% (collective use).

**Organon France**
106, Boulevard Haussmann. 75008 Paris
Tél Information médicale, Pharmacovigilance, Réclamations qualité, Qualité et Déontologie de l'information promotionnelle :
01 57 77 32 00
E-mail : info.medicale.fr@organon.com
